# Glycoproteomic and Single-Protein Glycomic Analyses Reveal Zwitterionic N-Glycans on Natural and Recombinant Proteins Derived From Insect Cells

**DOI:** 10.1016/j.mcpro.2025.100981

**Published:** 2025-05-05

**Authors:** Shi Yan, Jorick Vanbeselaere, Callum Ives, David Stenitzer, Lena Nuschy, Florian Wöls, Katharina Paschinger, Elisa Fadda, Johannes Stadlmann, Iain B.H. Wilson

**Affiliations:** 1 Institut für Biochemie, Universität für Bodenkultur, Wien, Austria; 2 Institut für Parasitologie, Veterinärmedizinische Universität, Wien, Austria; 3 Department of Chemistry, Maynooth University, Maynooth, Ireland; 4 School of Biological Sciences, University of Southampton, United Kingdom

**Keywords:** baculovirus, fucose, glucuronic acid, insect cells, N-glycans, phosphorylcholine

## Abstract

Insect cells are a convenient cell factory to produce recombinant glycoproteins. Their glycosylation potential is believed to be simple, needing primarily addition of glycosyltransferases to humanize the recombinant products. In this study, the native glycoproteome of *Spodoptera frugiperda* Sf9 and *Trichoplusia ni* High Five cells, examined using an LC-MS/MS approach, revealed not only which proteins are N-glycosylated but also indicated that the N-glycomes contain novel glucuronylated and phosphorylcholine-modified glycans, in addition to typical oligomannosidic and fucosylated structures. These data were corroborated by a parallel MALDI-TOF MS/MS analysis of N-glycosidase-released oligosaccharides. Molecular modeling analysis of one endogenous Sf9 glycoprotein correlated the occurrence of complex and oligomannosidic N-glycans with the accessibility of the occupied N-glycosylation sites. Further, we showed that the N-glycans of influenza hemagglutinins and SARS-CoV-2 spike glycoprotein produced in *Spodoptera* cells possess a number of glycan structures modified with phosphorylcholine, but core difucosylation was minimal; in contrast, the *Trichoplusia*-produced hemagglutinin had only traces of the former type, while the latter was dominant. Detection of phosphorylcholine on these glycoproteins correlated with binding to human C-reactive protein. In conclusion, not just oligomannosidic or truncated paucimannosidic N-glycans, but structures with immunogenic features occur on both natural and recombinant glycoproteins derived from insect cell lines.

## Highlights

• The HighFive and Sf9 insect cell lines express unusual glycan epitopes.
• Species-specific glyco-variations with glucuronic acid, fucose, and phosphorylcholine.
• N-glycans with phosphorylcholine present on insect cell-derived recombinant proteins.

## Graphical Abstract

**Figure:**
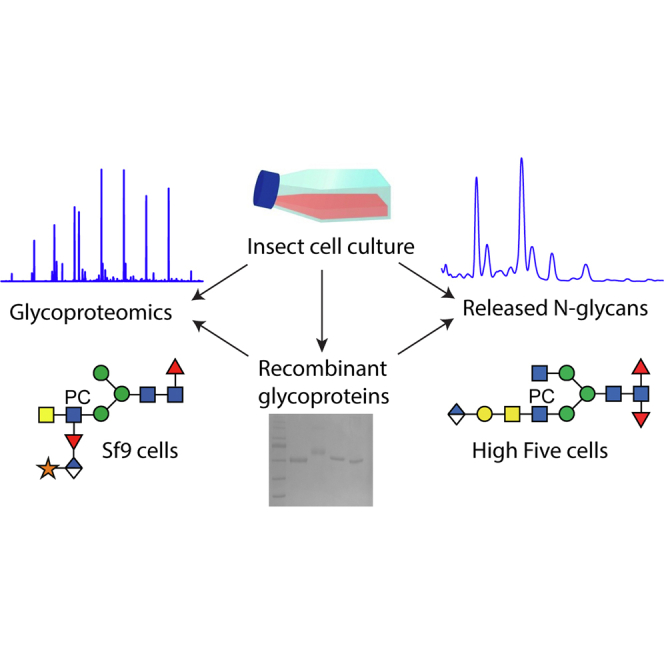

## In Brief

Insect cell lines are frequently used for expression of recombinant glycoproteins, including vaccines. Using two independent approaches, we show that the endogenous N-glycomes of High Five and Sf9 cells contain species-specific anionic N-glycan structures with different combinations of phosphorylcholine, fucose, hexuronic acid and/or pentose. Glycans with phosphorylcholine and fucose epitopes were also found on influenza hemagglutinin and SARS-CoV-2 Spike expressed in either cell line. Our data highlight the need for open search strategies for identifying glycans present on recombinant glycoproteins from non-mammalian sources.

As glycans are important for the proper folding of glycoproteins in the eukaryotic secretory pathway as well as for a range of bioactivities, production of recombinant antibodies, protein hormones, or vaccines for therapeutic use or for other *in vitro* purposes requires a glycosylation pattern which does not interfere with the intended application. Although mammalian cell lines are often used, production costs and other factors drive the search for other expression systems. Thereby, insect cells have been intensively studied as potential eukaryotic “cell factories”; however, differences in the glycan structures in insects have led to efforts to re-engineer their glycosylation machinery, for example, by introduction of mammalian glycosyltransferases (1). Generally, the asparagine-linked oligosaccharides (N-glycans) of insects are considered to be truncated with the degree of double core α1,3/α1,6-fucosylation differing between cell lines (2); core α1,3-fucose is known to be immunogenic in mammals as shown by the ability to raise antibodies against it in rabbits (3) or its binding to IgE from allergic patients (4). Nevertheless, even mammalian cell lines can produce antigenic carbohydrate structures—the possibly most spectacular case being allergy resulting from the presence of α1,3-linked galactose on Cetuximab derived from murine NS0 cells (5), whereas *N*-glycolylneuraminic acid found in, *e.g.*, rodents and red meat is also a xenoantigen in humans (6).

In recent years, it has become apparent that insects and insect cell lines are capable of rather complex forms of N- and O-glycosylation, including a range of anionic and zwitterionic modifications, such as the addition of glucuronic acid, sulfate, phosphoethanolamine, and phosphorylcholine (7, 8, 9, 10, 11, 12, 13). At the same time, viral glycoproteins expressed in insect cell lines are now being used as recombinant vaccines (14, 15), whether it be influenza hemagglutinin or coronavirus spike glycoprotein, both of which are N-glycosylated (16, 17). As glycosylation analyses of these are often based on examination of glycopeptides rather than the released glycans, some aspects of the glycan diversity on these products meant for administration into humans may be missed due to the limits of analytical sensitivity or incomprehensive database matching.

In the current study, we have appraised the natural N-glycoproteome of *Trichoplusia ni* High Five (BTI-Tn-5B1-4 or Hi5) and *Spodoptera frugiperda* Sf9 cell lines as well as the N-glycans of one “homemade” hemagglutinin expressed in *T. ni* cells, three commercially available hemagglutinins produced in two different *S. frugiperda* cell lines (two of which are components of an older trivalent form of Flublok) and one recombinant Spike protein vaccine also expressed in *S. frugiperda* cells (marketed as Nuvaxovid). Our data demonstrate that insect cell lines used for baculovirus expression not only naturally have highly varied N-glycomes but that glycoproteins expressed in these cell lines display a range of N-glycan structures which can be recognized by components of mammalian innate immune systems or which have motifs capable of eliciting anti-glycan immune responses.

## Experimental Procedures

### Glycan Release and Analysis of Cell Lines

**Fig. 1:**
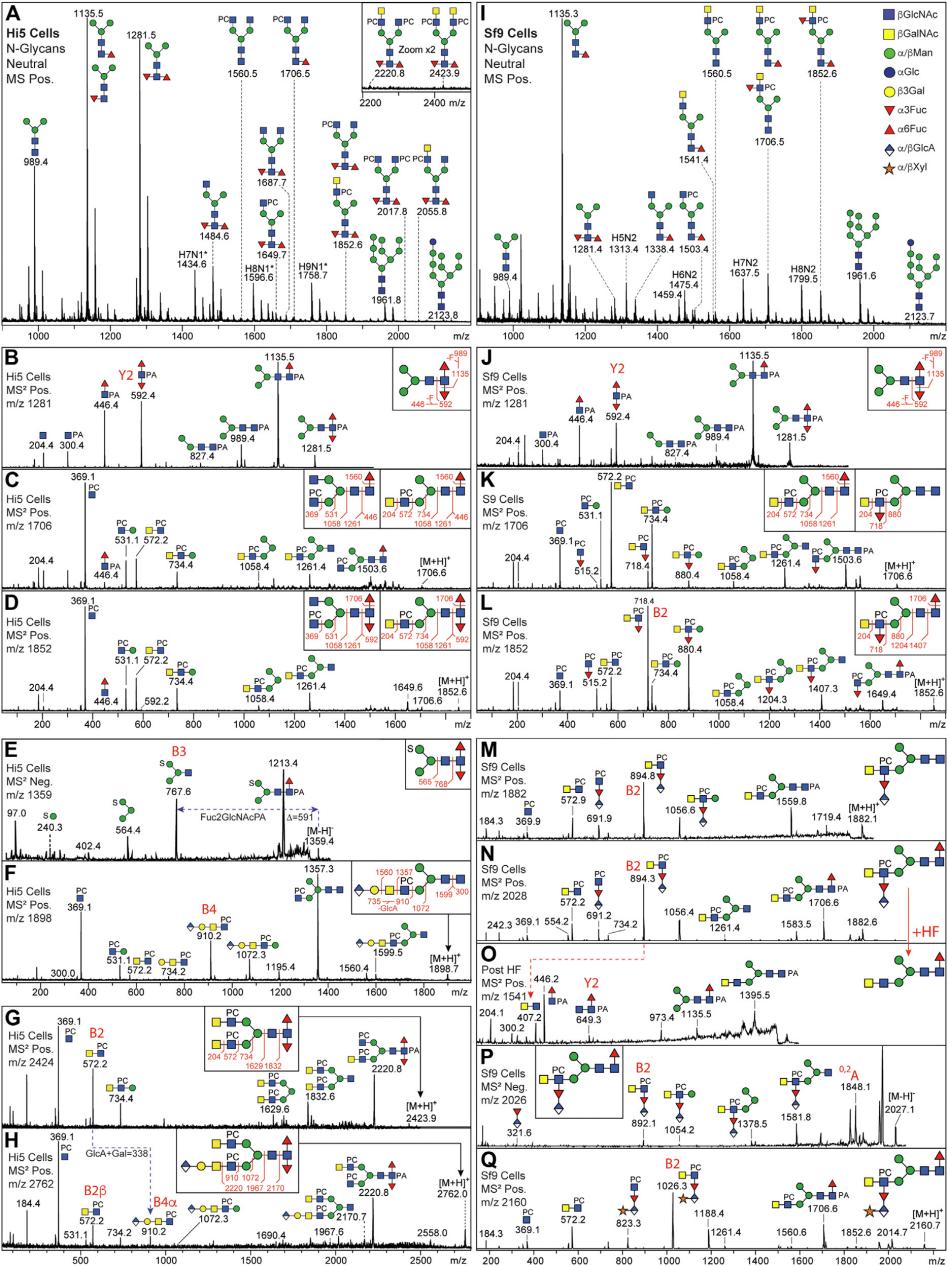
**Analysis of N-glycans of High Five and Sf9 insect-cell lines.** The pyridylaminated pools of N-glycans isolated from High Five and Sf9 cells were analysed by MALDI-TOF MS (*A* and *I*) and MS/MS (*B*–*D* and *J*–*L*); for low abundance glycans (*E*–*H* and *M*–*Q*), MS/MS was performed after RP-amide HPLC purification. The annotated *m/z* values are for [M+H]^+^ in the positive ion mode (MS Pos.) or [M-H]^-^ in the negative ion mode (MS Neg.). Glycans with both glucuronic acid and phosphorylcholine were detected in both cell lines but had contrasting structural motifs (see also Supplemental Figs. S1–S6 for further MS/MS as well as the effect of corroboratory enzymatic or chemical treatments). The effect of hydrofluoric acid treatment on the *m/z* 2028 glycan from Sf9 cells (compare *N* and *O*) and the negative mode MS/MS spectrum (*P*) are indications for a novel Fuc_1_GlcA_1_Xyl_1_ motif (see also Supplemental Fig. S5). Key fragments are annotated on peaks, and overall parent ion fragmentation patterns are drawn in boxes according to the Symbol Nomenclature for Glycans (see also key) (89), whereby PA, PC, and S indicate 2-aminopyridine, phosphorylcholine, and sulfate.

Approximately 10^7^ cells (ca. 0.5 g wet weight) of *S. frugiperda* Sf9 (CRL-1711) and *T. ni* High Five (BTI-TN5B1-4) were grown in HyClone media supplemented by 2% FBS. Cells were washed in PBS, boiled, and lysed; the resulting glycoprotein extracts were digested with thermolysin in 0.1 M ammonium bicarbonate at pH 8.0 and 70 °C for 2 h. Resulting glycopeptides were purified by cation exchange and desalted by gel filtration. Their N-glycans were released by recombinant PNGase Ar and endoglycosidase H (New England Biolabs) and purified by cation exchange, non-porous graphitized and C18 columns prior to subjected to reductive amination by 2-aminopyridine (PA) as in previous studies (7). The pyridylaminated neutral and anionic pools of N-glycans were then analyzed by MALDI-TOF MS/MS (Fig. 1); the N-glycomes were also fractionated by HPLC using an Ascentis Express 2.7 μ RP-Amide column (150 × 4.6 mm; Sigma-Aldrich) calibrated in terms of glucose units (g.u.) with an oligomaltose standard; glycans were eluted with a gradient of methanol in 100 mM ammonium acetate, pH 4, and detected by fluorescence at 320/400 nm (excitation/emission) (7). Selected fractions were subject to treatment with (i) hydrofluoric acid (48%) on ice for 48 h prior to evaporation, thereby cleaving α1,3-fucose and phosphorylcholine linkages or (ii) either jack bean α-mannosidase (New England Biolabs), jack bean β-hexosaminidase (Sigma), *Caenorhabditis elegans* HEX-4 β-*N*-acetylgalactosaminidase (produced in house (18)), a deep-sea sediment metagenome-derived endo-β-N-acetylgalactosaminidase (NgaDssm (19); kind gift of Dr Tomomi Sumida), bovine α-fucosidase (Sigma) or human β-glucuronidase (biotechne) for 3 hours or overnight at 37 °C. MALDI-TOF MS/MS was performed using either an Autoflex Speed or a Rapiflex (Bruker Daltonics) instrument in positive and negative reflectron modes with 6-aza-2-thiothymine (ATT; Alfa-Aesar, Thermo Scientific) as matrix. MS/MS was performed by laser-induced dissociation of the [M+H]^+^ or [M-H]^-^ molecular ions. With the Autoflex Speed, typically 1000 shots were summed for MS (reflector voltage, lens voltage, and gain of 27 kV, 9 kV, and 2059 V, respectively) and 5000 for MS/MS (reflector voltage, lift voltage, and gain of 27 kV, 19 kV, and 2246 V, respectively); for the Rapiflex, typically 4000 shots were summed for MS (reflector voltage, lens voltage, and gain of 20.8 kV, 11.6 kV, and 1909 V, respectively) and 10,000 to 20000 for MS/MS (reflector voltage, lift voltage, and gain of 23.8 kV, 19 kV, and 2171 V, respectively). Spectra were processed with the manufacturer’s software (Bruker Flexanalysis 3.3.80) using the SNAP algorithm with a signal/noise threshold of six for MS (unsmoothed) and three for MS/MS (smoothed four times). Glycan spectra were manually interpreted on basis of the masses of the predicted component monosaccharides, fragmentation patterns, differences of mass in glycan series and comparison with coeluting structures from insects or nematodes. Assigned glycans had an interpretable MS/MS spectrum with at least three fragment ions, including a Y1-ion (20) at *m/z* 300, 446, and/or 592 corresponding to a pyridylaminated reducing core GlcNAc_1_Fuc_0-2_ or (in the case of phosphorylcholine-modified glycans) B ions at *m/z* 369 and 572 (*i.e.*, HexNAc_1-2_PC_1_). Lists of theoretical *m/z* values for the predicted glycan compositions are presented in Supplemental Tables S1 and S2.

### Protein Extraction, Tryptic Digestion, and Glycopeptide Enrichment

Sf9 cells (CRL-1711, ATCC, Manassas) and High Five cells (BTI-TN5B1-4, Invitrogen Corp) were grown at 27 °C in IPL-41 medium (Sigma) supplemented with 5% foetal bovine serum (Gibco, Thermo Fisher Scientific, Massachusetts), 0.01% penicillin and streptomycin, as well as 2% yeast extract (Gibco, Thermo Fisher Scientific). Both cell lines were grown to confluency in T-75 flasks. For glycopeptide preparation, both cell lines were cultivated in IPL-41 medium, without further additives, for 4 days at 27 °C, and were harvested by scraping.

Cells were washed with phosphate-buffered saline, pelleted by centrifugation (5000*g*, 10 min), and lysed by the addition of 1 ml 8 M urea in 10 mM hydrochloric acid, pH 3. Prior to reduction (20 mM dithioerythritol, 30 min, at 90 °C) and alkylation (40 mM iodoacetamide, 60 min, at room-temperature, in the dark) of proteins, total protein concentration of the lysates were determined by Micro BCA Protein Assay Kit (Thermo) and their pH values were adjusted to pH 8.0 by the addition of 100 μl 1 M TRIS/HCl-buffer (pH 8.0). Trypsin Gold (mass spectrometry grade, Promega) was added in a protein-to-trypsin ratio of approximately 100:1, and proteolytic digestion was allowed to proceed at 37 °C, for 3 h, under constant shaking at 700 rpm. After this, trypsin activity was quenched by adjusting the samples to pH 2 by the addition of 10% trifluoracetic acid (TFA). Subsequently, the acidified protein digests were loaded onto methanol-primed and conditioned (0.1% TFA) reversed-phase solid-phase cartridges (C18-SPE, 200 mg; Waters), washed with 0.1% TFA acid, and (glyco)peptides were eluted in 1.2 ml 50% acetonitrile, containing 0.05% TFA. The C18-SPE recovered (glyco)peptides were dried in a speed-vac concentrator. A single sample per cell line was generated by combining three technical replicates of the respective cultures.

Glycopeptides were enriched using ion-pairing hydrophilic interaction (IP-HILIC) chromatography, as reported previously (21). In brief, dried C18-SPE purified (glyco)peptides were re-suspended in 50 μl 80% acetonitrile, containing 1% TFA, and fractionated (fraction volume of 1 ml, each) using a TSK-Amide 80 column (4.6 × 250 mm, 5 micron, Tosoh), by developing a linear gradient from 80% acetonitrile, containing 0.1% TFA, to 50% acetonitrile, containing 0.1% TFA, at a flow-rate of 1 ml/min, over 30 min 15 IP-HILIC fractions were collected (mins. 25–39) and dried in a Speedvac concentrator.

### LC-ESI-MS/MS Analysis of Glycopeptides

The dried IP-HILIC fractions were resuspended in 20 μl 0.1% formic acid, subjected to reversed-phase chromatographic separation (nanoEase M/Z HSS T3 column, 100 Å, 1.8 μm, 300 μm × 150 mm, Waters) by developing a linear gradient from 0.8% acetonitrile in 0.1% formic acid, to 32% acetonitrile and 0.1% formic acid, at a flow-rate of 6 μl/min over 80 min, followed by a 5 min linear gradient from 32% acetonitrile and 0.1% formic acid to 76% acetonitrile and 0.1% formic acid. Data-dependent MS/MS analysis was performed by an Orbitrap Exploris 480 (Thermo) instrument, equipped with its standard H-ESI source operated in positive mode, using the following settings: MS data were acquired in the mass-range from 350 to 1500 at a resolution of 60,000, MS/MS data (isolation window 1.4 *m/z*; isolation offset 0.5 *m/z*, normalized AGC target 200%) were automatically acquired for (glyco)peptide precursor ions of charge-states two up to six and above a threshold of 800,000, using stepped HCD (normalized collision energies 28, 30 and 35) in centroid mode, with the first fixed mass at *m/z* = 120, at a resolution of 30,000.

### Data-analysis and Glycopeptide Identification

**Fig. 2:**
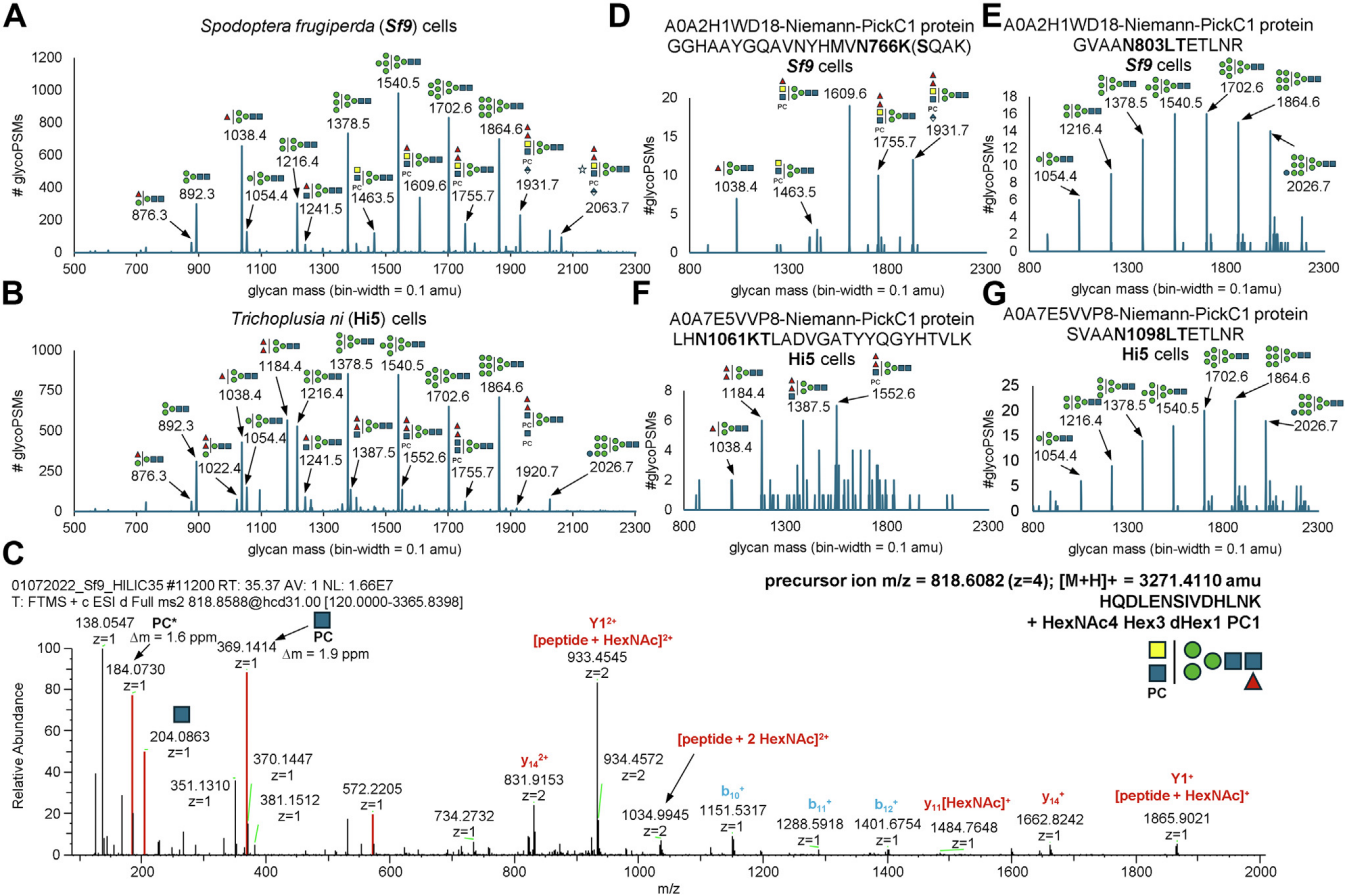
**Glycoproteomic characterization of Sf9 and High Five cells.** Whole cell lysates of insect cell lines were analyzed by data-dependent LC-ESI-MS/MS, and glycopeptides were identified using a glyco-dedicated “open-search” approach. Based on these data, semi-quantitative histograms (glycan mass bin-width = 0.1 amu) were reconstructed from spectral counting of automatically determined mass differences between the originally measured precursor and the Y1-fragment ions. The histograms confirmed the accumulation of specific glycan-masses (in amu) for (*A*) Sf9 and (*B*) High Five cells. Close inspection of (*C*) MS/MS spectra that contributed to unusual glycan-mass bins revealed the co-occurrence of specific, glycan-derived fragment ions that are highly indicative for phosphorylcholine-bearing *N*-acetylglucosamine (*i.e.* 184.1 amu, 369.1 amu, 572.2 amu); the example spectrum is for a glycopeptide derived from the Sf9 tetraspanin 1/CD63 homologue (Uniprot A0A2H1WWQ3). Semi-quantitative comparisons of site-specific glycosylation events within the NPC1 intracellular cholesterol transporter between Sf9 and High Five cells revealed that (*D*) N^766^ of *S. frugiperda* NPC1 was decorated with phosphorylcholine-containing complex-type N-glycans. *E*, N^803^ of *S. frugiperda* NPC1 was exclusively found decorated with oligo-mannose type N-glycans. *F*, N^1060^ in *T. ni* NPC1 is decorated with di-fucosylated N-glycans in High Five cells. *G*, N^1098^ in *T. ni* NPC1 was exclusively found decorated with oligo-mannose type N-glycans. Cartoons represent tentative structure assignments based on compositions (inferred from glycan mass) using the Symbol Nomenclature for Glycans (89).

Raw MS/MS data were extracted, refined (*i.e.* precursor mass and charge-state; no scan merging) and converted into the.mgf file-format using PEAKS X Pro Studio 10.6 (build 20,201,221). Subsequently, data were processed using custom-coded perl-scripts (“SugarQBits”; all scripts are freely available at http://homepage.boku.ac.at/jstadlmann) which allowed for an N-glycopeptide-specific “open-search” approach. In brief, of all charge-deconvoluted and deisotoped MS/MS data, only those containing the HexNAc-specific oxonium ion (*i.e.* 204.0867; mass-precision ±10 ppm) were retained and further analyzed for the co-occurrence and the intensities of three diagnostic fragment ion signals (mass-precision ±10 ppm), which potentially corresponded to the prominent glycopeptide Y1-fragment ion (*i.e.* [peptide+HexNAc+H]^+^, the neutral losses of 203.0794 amu and 120.0423 amu from the respective Y1-ion candidate). Of MS/MS spectra that concomitantly contained all three fragment ion signals, the MS/MS precursor mass information (*i.e.* stored in.mgf file-format as PEPMASS) was adjusted to the mass of the putative Y1-fragment ion detected (*i.e.* “SugarQBits_Kassonade.pl”) and a series N-glycan and PC-specific oxonium fragment ion signals (*i.e.* 128.0549,138.0550,144.0655, 163.0601, 168.0655, 186.0761, 204.0866, 243.0264, 274.0921, 290.0870, 292.1027, 308.0976, 323.2240, 366.1395, 184.0732, 351.1314, 369.1420, 531.1943 amu) were removed (*i.e.* “SugarQBits_RepX”; mass-precision ±10 ppm). Amino acid sequences of glycopeptides were identified from the pre-processed.mgf-files using Comet, implemented in SearchGUI (version: 4.1.11) (22), using species-specific proteome sequence data-based (*i.e.* UP000829999 for Sf9 cells, UP000322000 for High Five cells; uniprot.org), concatenated with their respective reversed sequences as decoys, and the following search engine settings: semi-tryptic digest, allowing up to one missed cleavage, carbamidomethylation as fixed modification of all cysteines, oxidation as variable modification to all methionine residues, HexNAc (including the neutral loss of 203.0794 amu) as variable modification to all serine, threonine, and asparagine residues. Spectral matching was performed with a mass precision of ±10 ppm on the precursor level, and ±0.05 amu at the MS/MS level. Contaminants were not considered or excluded. Glycopeptide identifications (search-engine rank 1, peptide length greater than 7, and at least one HexNAc-modified residue) were manually filtered to an approximately 1% false discovery rate at the spectrum level, using the target-decoy approach (23), as reported previously (21). No site localization of *N*-glycans within the peptides was performed, but a listing of probable glycosylation sites (Asn-Xaa-Ser/Thr/Cys) is given in the Supplemental Data File; while Asn-Xaa-Cys sites are predicted, no glycopeptide with only a Asn-Pro-Ser/Thr/Cys and no other Asn-Xaa-Ser/Thr/Cys was assigned. GO-term analysis was performed for the respective glycoprotein sets versus all protein sequences identified in this study as species specific reference sets using Blast2Go (version 6.0.3 - build 202109151544) (24). Glycan-mass histograms (Fig. 2, *A* and *B*) were constructed from the automatically (*i.e.*, SugarQBits_Kassonade) determined mass-differences between the original glycopeptide precursors and the identified peptide-sequences of all glyco-PSMs (FDR <1%), at a bin-width of 0.1 amu, by spectral counting. For glycosylation, site-specific glycan-mass histograms (Fig. 2, *D*–*G*), only glyco-PSMs (FDR <1%) covering the respective site were counted.

### Molecular Modeling

The 3D structure of *S. frugiperda* NPC1 was obtained from the AlphaFold Protein Structure Database (25) using UniProt ID A0A2H1WD18 (26). The termini of the protein (residues 1–60 and 1026–1043) were truncated due to low pLDDT scores, indicating poor model confidence. The protein structure was glycosylated at N^175^, N^327^, N^700^, N^766^, and N^803^ with a Man_5_GlcNAc_2_ oligomannose glycan (GlyTouCan ID: G00028MO) using a structure from the GlycoShape Glycan Database (27), and attached using the Re-Glyco tool of the GlycoShape platform. The protein was embedded in a POPC bilayer and solved with water molecules and 150 mM NaCl using PACKMOL-Memgen (28). The simulation system was parameterized using the tleap module of AmberTools (29). The charged N- and C-terminal residues were neutralized by capping with ACE and NME groups, respectively. The AMBER 14SB force field (30) was used to model proteins and ions, the GLYCAM06j-1 (31) force field was used to model glycans, the Lipid21 force field (32) was used to model lipid molecules, and the TIP3P water model was used to model solvent molecules (33).

The energy of the system was minimized using the steepest descent algorithm, with all atoms of the protein, lipid head groups, and glycans restrained. The system was then equilibrated in the NVT ensemble, with the system gradually heated from 0 to 100 K, and then from 100 K to 300 K. The system was then equilibrated in the NPT ensemble to maintain the pressure at 1 bar. Position restraints placed on the atoms of the protein, lipid head groups, and glycans were gradually released during the equilibration process. The temperature was maintained at 300 K using Langevin dynamics with a collision frequency of 1 ps-1, and the pressure was maintained at 1 bar using semi-isotropic position scaling with a Berendsen barostat and a pressure relaxation time of 1 ps. Periodic boundary conditions were used throughout the simulations. The van der Waals interactions were truncated at 11 Å, and Particle Mesh Ewald (PME) was used to treat long-range electrostatics with B-spline interpolation of order 4. The SHAKE algorithm was used to constrain all bonds containing hydrogen atoms and to allow the use of a 2 fs time step for all simulations.

The system was then simulated in triplicate for 600 ns each, with the first 100 ns discarded as additional equilibration. Therefore, all analyses reported are from a total of 1.5 μs of uncorrelated simulations. All simulations were performed using AMBER18 (34) on resources provided by the Irish Centre for High-End Computing (ICHEC).

### Recombinant Proteins

Four preparations of three different influenza A hemagglutinins were used in the study: soluble A/California/04/2009 (H1N1), either soluble His-tagged expressed in *T. ni* Tnms42 cells (kind gift of Dieter Palmberger, Universität für Bodenkultur Wien; Tnms42 is an alpha-nodavirus-free cell line generated as a subclone of High Five cells) or full-length C-terminally His-tagged expressed in *S. frugiperda* Sf9 cells (purchased from Genscript, Piscataway, NJ, USA); A/California/07/2009 (H1N1) full length expressed in *S. frugiperda* ExpresSF+ cells (purchased from Protein Sciences Corporation); A/Victoria/361/2011 (H3N2) full length expressed in *S. frugiperda* ExpresSF+ cells (also purchased from Protein Sciences Corporation). For analyses of the SARS-CoV-2 Spike glycoprotein, the original Wuhan-1 full-length sequence expressed in Sf9 cells was used (NVX-CoV237, *i.e.*, Nuvaxovid; Novavax). A summary of analyses of these samples is shown in Supplemental Table S3.

### Western Blotting

Before SDS-PAGE, proteins were precipitated (mixed with a 5-fold volume excess of methanol), incubated at −80 °C for 1 h, centrifuged at 4 °C, 21,000*g* and dissolved in a reducing sample buffer. After electrophoresis (10 μg/lane) and blotting to a nitrocellulose membrane, the following reagents for detection of glycan epitopes were employed: anti-horseradish peroxidase antibody (Sigma-Aldrich; 1:10,000 diluted in Tris buffered saline with 0.05% Tween and 0.5% BSA, to detect core α1,3-fucose (3, 35), C-reactive protein (MP Biochemicals, Santa Ana; 1:200, to detect phosphorylcholine (36, 37) or biotinylated *Aleuria aurantia* lectin (Vector Labs; 1:1000, to detect fucose (35, 36) followed by the relevant alkaline phosphatase-conjugated secondary antibodies and colour development with SigmaFAST BCIP/NBT (7).

### Glycan Release and Analysis of Glycoproteins

Purified recombinant proteins were subject to SDS-PAGE and the bands were excised, dried, washed and alkylated prior to addition of trypsin; the tryptic peptides were then heat-inactivated and isolated prior to MALDI-TOF MS analysis using α-cyano-4-hydroxycinnamic acid (ACH) as matrix; the peptides were then treated with recombinant PNGase A and the glycans purified using solid phase extraction (7). Released N-glycans were reductively labeled using 2-aminopyridine and fractionated by HPLC using an Ascentis Express 2.7 μ RP-Amide column (150 × 4.6 mm; Sigma-Aldrich); glycans were eluted with a gradient of methanol in 100 mM ammonium acetate, pH 4 and detected by fluorescence at 320/400 nm (excitation/emission) (7). Monoisotopic MALDI-TOF MS was performed as described earlier for the N-glycomes.

### Proteomic Analysis of Hemagglutinins

Protein bands, also post PNGase F treatment, for the full-length A/California/04/2009 were cut from the SDS-PAGE gel (∼15 μg/lane). After washing and destaining, proteins were fixed in the gel and reduced with dithiothreitol and alkylated with iodoacetamide. In-gel digestion was performed with trypsin (Trypsin Gold, Mass Spectrometry Grade; Trypsin-ultra, MS grade, New England Biolabs) with a final trypsin concentration of 20 ng/μl in 50 mM aqueous ammonium bicarbonate and 5 mM CaCl_2_ either for 8 h or overnight at 37 °C. Afterward, peptides were extracted thrice with 50 μl of 5% trifluoroacetic acid in 50% aqueous acetonitrile supported by ultrasonication for 10 min. Extracted peptides were dried down in a vacuum concentrator (Eppendorf, Hamburg, Germany) and resuspended in 0.1% trifluoroacetic acid for LC-MS/MS analysis. Peptides were separated on a nano-HPLC Ultimate 3000 RSLC system (Dionex). Sample pre-concentration and desalting were accomplished with a 5 μm Acclaim PepMap μ-Precolumn (300 μm inner diameter, 5 μm, 100 Å; Dionex). For sample loading and desalting 2% acetonitrile in ultra-pure H_2_O with 0.05% trifluoroacetic acid was used as a mobile phase with a flow rate of 5 μl/min. Separation of peptides was performed on a 25 cm Acclaim PepMap C18 column (75 μm inner diameter, 2 μm, 100 Å) with a flow rate of 300 nl/min. The gradient started with 4% B (80% acetonitrile with 0.08% formic acid) for 7 min, increased to 31% in 30 min, and to 44% in an additional 5 min. It was followed by a washing step with 95% B. The mobile phase A was ultra-pure H_2_O with 0.1% formic acid. For mass spectrometric analysis, the LC was directly coupled to a high-resolution Q Exactive HF Orbitrap mass spectrometer. MS full scans were performed in the ultrahigh-field Orbitrap mass analyzer in ranges *m/z* 350 − 2000 with a resolution of 60,000, the maximum injection time (MIT) was 50 ms, and the automatic gain control (AGC) was set to 3 × 10^6^. The top 10 intense ions were subjected to Orbitrap for further fragmentation *via* high-energy collision dissociation (HCD) activation over a mass range between *m/z* 200 and 2000 at a resolution of 15,000 with the intensity threshold at 4 × 10^3^. Ions with charge state +1, +7, +8, and >+8 were excluded. Normalized collision energy (NCE) was set at 28. For each scan, the AGC was set at 5 × 10^4^ and the MIT was 50 ms. Dynamic exclusion of precursor ion masses over a time window of 30s was used to suppress repeated peak fragmentation.

For glycoproteomic data analysis, a pre-released FragPipe v21.1 build-21 using MSFragger v4.1-rc21 and Philosopher v5.1.1-RC12 was used to identify N-glycopeptides (38, 39, 40). The “glyco-N-HCD” workflow was selected with default setting (*i.e.*, one trypsin missed cleavage allowed, cysteine carbamidomethylation as fixed modification, methionine oxidation as variable modification, HexNAc—including its neutral loss—as variable modification of N-glycosites, peptide and glycan FDR are set at 1%) but with the addition of PC related oxonium ions (*e.g.*, 184.0728, 351.1310, 369.1416, 531.1950, 572.2210, and 734.2738 amu) in MSFragger (*Glyco/Labile Mods* sub-tab) and PTMs (*Diagnostic feature extraction* sub-tab) which already contained a series of N-glycan specific oxonium ions (*e.g.* 128.0549, 138.0550, 144.0655, 163.0601, 168.0655, 186.0761, 204.0866, 243.0264, 274.0921, 290.0870, 292.1027, 308.0976, 323.2240, and 366.1395 amu). Spectral matching was performed with a mass-precision of ±20 ppm on the precursor level, and ±20 ppm at the MS/MS level. Also, PTM-Shepherd was set on “Glyco Search” with a custom glycan database including 49 entries corresponding to the list of Hi5/Sf9 N-glycans identified in this study. The glycan databases folder (only available in the pre-release version) was accordingly modified to allow the engine recognition of GlcA and PC residues.

### Experimental Design and Statistical Rationale

For this qualitative study, one sample of each cell line or glycoprotein was used. As no further statistical analyses were performed on the glycoproteomic or glycomic datasets, no controls or randomization methods were applied.

## Results

### MALDI-TOF MS Analyses of the N-Glycomic Capacity of High Five and Sf9 Cells

We have previously analyzed the N-glycome of High Five cells (9) but not of Sf9 cells. Indeed, there appears to have been no analyses of the overall N-glycan profile of the latter, only of the Sf21 derivative (2), although Sf9 cells are frequently used for recombinant protein expression. In the present study, to concentrate on the more processed structures, we used PNGase Ar and Endo H for the release of the N-glycans from both cell lines. The relative dominance of the difucosylated MMF^3^F^6^ in High Five cells (Fig. 1, *A* and *B*) is as expected (9), but an obvious, nevertheless low, signal for this glycan in Sf9 cells was also observed (Fig. 1, *I* and *J*). In addition, phosphorylcholine-modified N-glycans were found in both cell lines (Fig. 1, *C*, *D*, *K*, and *L* and Supplemental Figs. S1 and S2); this is in accordance with previous analyses of two Lepidopteran species (9), whereby underlying LacdiNAc and fucosylated LacdiNAc motifs were proven by enzymatic digestion (Supplemental Fig. S2) and the phosphorylcholine modification is associated with intense B ions, *e.g*., at *m/z* 369, 515, 572 and 718 (HexNAc_1-2_Fuc_0-1_PC_1_). Sulfated paucimannosidic structures were also detected in High Five cells (Fig. 1*E* and Supplemental Fig. S1*C*); based on MS/MS and glycosidase digestion data, sulfation was concluded to occur on either α-mannose or core α1,6-fucose residues (Fig. 1 and Supplemental Figs. S1 and S3), compatible with previous data on N-glycans from other insects (11, 13). Sulfated glycans are only observed in negative ion mode, and those from lepidopteran sources are resistant to hydrofluoric acid treatment (9), thus distinguishing them from potential isobaric phosphorylated structures (41).

In High Five cells, N-glycans of 1800 to 3000 Da carrying one or two phosphorylcholine residues and a difucosylated core motif with glucuronic acid were detected, also upon HPLC fractionation, displaying dominant B-fragments at *m/z* 572 and 910 corresponding to HexNAc_2_PC_1_ and HexNAc_2_Hex_1_PC_1_HexA_1_ (Fig. 1, *F*–*H* and Supplemental Figs. S1*D* and S4). The possibility that the 176 Da modification is monomethylated hexose, which is isobaric with glucuronic acid, is negated by the detection of the glycans in negative ion mode, and their sensitivity to human β-glucuronidase. There were two glucuronylated structures resistant to this treatment (*m/z* 2597 and 2965) with MS/MS fragments at *m/z* 1113 (HexNAc_3_Hex_1_PC_1_HexA_1_), suggestive of a HexNAc-substitution of the glucuronic acid (Supplemental Fig. S4*M*).

Unusual masses of over 1800 Da were also detected in the Sf9 anionic pool, also as predicted from the glycoproteomics data described below, but these did not correspond to any of the larger structures in High Five cells. These masses were detected in both positive and negative modes (*m/z* 1882, 2014, 2028 and 2160 as [M+H]^+^; Supplemental Fig. S5). MS/MS revealed novel fragmentation patterns with dominant B-fragments at *m/z* 894 and 1026 (Fig. 1 *M–Q*); based on the *Δm/z* series, these fragments are predicted to correspond to HexNAc_2_Fuc_1_PC_1_HexA_1_Pnt_0-1_. This is corroborated by the stepwise loss upon hydrofluoric acid treatment of 165 Da (*i.e.*, phosphorylcholine) and 322 or 454 Da (*i.e.*,. Fuc_1_HexA_1_Pnt_0-1_), as shown by negative and positive ion mode MALDI-TOF-MS (Supplemental Fig. S5). Thus, based on the MS/MS data, the detection in both MS modes and the relationship to the “simple” *m/z* 1706 and 1852 glycans (Fig. 1), the predicted compositions for these glycans are Hex_3_HexNAc_4_Fuc_1-2_HexA_1_Pnt_0-1_PC. Considering the monosaccharide building blocks known from insect glycoconjugates, it is likely that the hexuronic acid is glucuronic acid and the pentose is xylose; the substitution of antennal fucose by glucuronic acid has been previously observed for permethylated N-glycans from a mollusk (42), whereas XylGlcA is the basic repeating unit of matriglycan (43). Lists of theoretical masses for all identified glycans are given in Supplemental Tables S1 and S2 and a summary of species-specific motifs is shown in Supplemental Fig. S6.

### LC-MS-Based Glycoproteomic Analyses of Sf9 and High Five Cells

Independent of the glycomic analyses, we analyzed glycopeptide-enriched IP-HILIC fractions of Sf9 and HighFive whole cell lysates by data-dependent LC-ESI-MS/MS. To enable a strictly data-driven and glycome-information independent glycoproteomics data-analysis approach, we developed a suite of scripts (all implemented in the cross-platform programming language Perl) that do not require the input of a glycan database or mass list (21, 44, 45, 46). In a first step, the results of our glyco-dedicated “open-search” approach allowed us to extract automatically determined mass-differences between the originally measured precursor masses of potential N-glycopeptides and a potential Y1-fragment ion mass (“delta-masses”), for each MS/MS spectrum in both datasets. Based on these data we re-constructed semi-quantitative “glycan-mass” histograms (Fig. 2*A*), which closely resemble MALDI-ToF-MS spectra. These histograms confirmed the accumulation of specific MS/MS spectra delta-masses (Fig. 2, *A* and *B*) for both cell lines. Intriguingly, the masses of these most densely populated glycan-mass bins corresponded to theoretical masses of known N-glycan compositions, such as the oligo-mannose type N-glycans (*i.e.* ranging from Hex_4_HexNAc_2_ to Hex_10_HexNAc_2_) or small, core-fucosylated paucimannosidic N-glycans (*e.g.*, Hex_3-4_HexNAc_2_Fuc_1_). Further, semi-quantitative comparison of all major “glycan-mass” bins detected in Sf9 and High Five cells, however, also highlighted important differences between the two data-sets. While High Five cells were found to abundantly exhibited glycan-masses that corresponded to previously described, insect-specific di-fucosylated N-glycan compositions (*i.e.* Hex_3_HexNAc_2-3_Fuc_2_), the reconstructed N-glycome of Sf9 cells was found to also contain higher molecular weight glycans of non-canonical composition (*e.g.* 1609.5, 1755.5, 1785.6, 1931.7 and 2063.7 amu). Close inspection of the MS/MS spectra that contributed to these populations of unusual glycan-mass bins (Fig. 2*C*) revealed the co-occurrence of specific, glycan-derived fragment ions that are highly indicative for phosphorylcholine-bearing *N*-acetylglucosamine (*e.g.* 184.1 amu, 369.1 amu, 572.2 amu). Considering also the MALDI-TOF MS/MS data for released N-glycans as well as the *Δm/z* of 146, 176 and 132, suggestive of fucose, hexuronic acid and pentose modifications, the identification of phosphorylcholine as a constituent of these unsual glycan masses led us to deduce their potential compositions as Hex_3_HexNAc_4_Fuc_1-2_PC, Hex_3_HexNAc_4_Fuc_1-2_HexA_1_PC and Hex_3_HexNAc_4_Fuc_2_HexA_1_Pnt_1_PC.

In a next step, we used a generic proteomics MS/MS search engine, that is, Comet implemented in SeachGUI (22, 47), to identify the peptide sequences of the individual glycopeptides. From the MS/MS data pre-processed by our pipeline, the search engine—merely including N-acetylhexosamine as a variable modification to every asparagine—provided the identification of 1680 unique glycopeptide amino-acid sequences from Sf9 cells, and 1721 unique glycopeptide amino-acid sequences from High Five cells, which derived from at least 666 and 621 glycoproteins, respectively. From this, we then queried the glycoproteomes of Sf9 and HighFive cells, glycan mass specifically. We compiled glycoprotein groups based on the detection of specific N-glycan masses and performed GO-term enrichment analysis (Supplemental Fig. S7). For both cell lines, this analysis confirmed the enrichment of ER-resident proteins within the group of oligomannose (*i.e.* Hex_5-10_HexNAc_2_) bearing glycoproteins. In contrast, for both datasets, the populations of glycoproteins found to carry paucimannosidic and complex-type N-glycans (*i.e.*, Hex_3-4_HexNAc_2-3_Fuc_1_ and Hex_3_HexNAc_4_Fuc_1-2_PC) were markedly enriched for plasma membrane and cell surface-located proteins.

More importantly, however, our glycoproteomic datasets also allowed for semi-quantitative comparisons of site-specific glycosylation events. For example, we compared the N-glycosylation profiles of two closely spaced asparagine residues within the NPC1 intracellular cholesterol transporter (48) of Sf9 and HighFive cells (Fig. 2, *D*–*G*; Uniprot entries A0A2H1WD18 and A0A7E5VWB4/A0A7E5VVP8). Although N^803^ from *S. frugiperda* and the corresponding N^1098^ from *T. ni* NPC1 were exclusively decorated with oligo-mannose type N-glycans, we found markedly different glycan populations linked to N^766^ from *S. frugiperda* and N^1061^ from *T. ni*: while Sf9 cells predominantly glycosylated N^766^ with phosphorylcholine-containing complex-type N-glycans (*i.e.* Hex_3_HexNAc_4_Fuc_1-2_PC and Hex_3_HexNAc_4_Fuc_2_HexA_1_PC), the corresponding site is decorated with di-fucosylated N-glycans (*i.e.* Hex_3-4_HexNAc_2_Fuc_2_ and Hex_3_HexNAc_4_Fuc_2_PC) in HighFive cells.

In terms of glycosylation sequons, although we considered any Asn, Ser or Thr in the entire Hi5 proteome as a potential (N or O-) glycosylation site, 88% (*i.e.* 745 of 846) of unique glycopeptide sequences comprised at least one of the established N-glycosylation site consensus motifs, without P for X. Furthermore, 33 glycopeptide sequences did not contain any Asn residue whatsoever and were thus deemed O-glycopeptides. 47 of the remaining 68 putative N-glycopeptide sequences were found to contain Asn residues at the penultimate C-terminal position of their sequence and were manually confirmed to contain an established N-glycosylation motif within the context of the extended protein sequences. Only in 21 of all unique glycopeptide sequences (of 846; *i.e.*, 2.5%), we could not identify an N-glycosylation motif. These doubtful glycopeptide sequences represented 0.4% of all glyco-PSMs (*i.e.*, of 9312), well below our 1% FDR at the glyco-PSM level (this is a glycopeptide FDR and not a glyco-site FDR). Similar numbers were obtained for the Sf9 glycoproteomics dataset, for which 34 of 776 unique glycopeptide sequences possess a penultimate Asn residue within an N-glycosylation motif in the context of the extended protein sequence and a further 30 contain no Asn residue, while generally containing at least one Ser/Thr residue and so potentially O-glycosylated; a final set of 17 were doubtful as they contain no canonical N-glycosylation motif. Listings of Asn-Xaa-Ser/Thr/Cys sequences are given in the Supplementary Excel file.

#### Molecular Modeling Data

The glycoproteomics data analysis of the NPC1 from Sf9 and High Five cells shows an interesting diversity in the type of N-glycosylation at different sites. We used all-atom molecular dynamics (MD) simulations to investigate if this heterogeneity could be reconciled with the diversity in the protein landscape and with the specific location of the sites relative to the lipid bilayer, and thus their accessibility by glycosylhydrolases and glycosyltransferases. To achieve this goal, we reconstructed a 3D model of NPC1 embedded in a simple POPC bilayer, with the protein structure (UniProt ID A0A2H1WD18) obtained from the AlphaFold Protein Structure Database (25), as no experimentally-determined structures of NPC1 were available at the time of writing. The N-glycosylation sites at N^175^, N^327^, N^700^, N^766^, and N^803^ were occupied with a Man_5_GlcNAc_2_ oligomannose N-glycan (GlyTouCan ID: G00028MO), as the key precursor for the functionalization of N-glycans to all complex types. The Man_5_GlcNAc_2_ structures were sourced from the GlycoShape Glycan Database (27), and linked to the corresponding Asn using the Re-Glyco tool available on the GlycoShape platform (https://glycoshape.org/Re-Glyco).

**Fig. 3:**
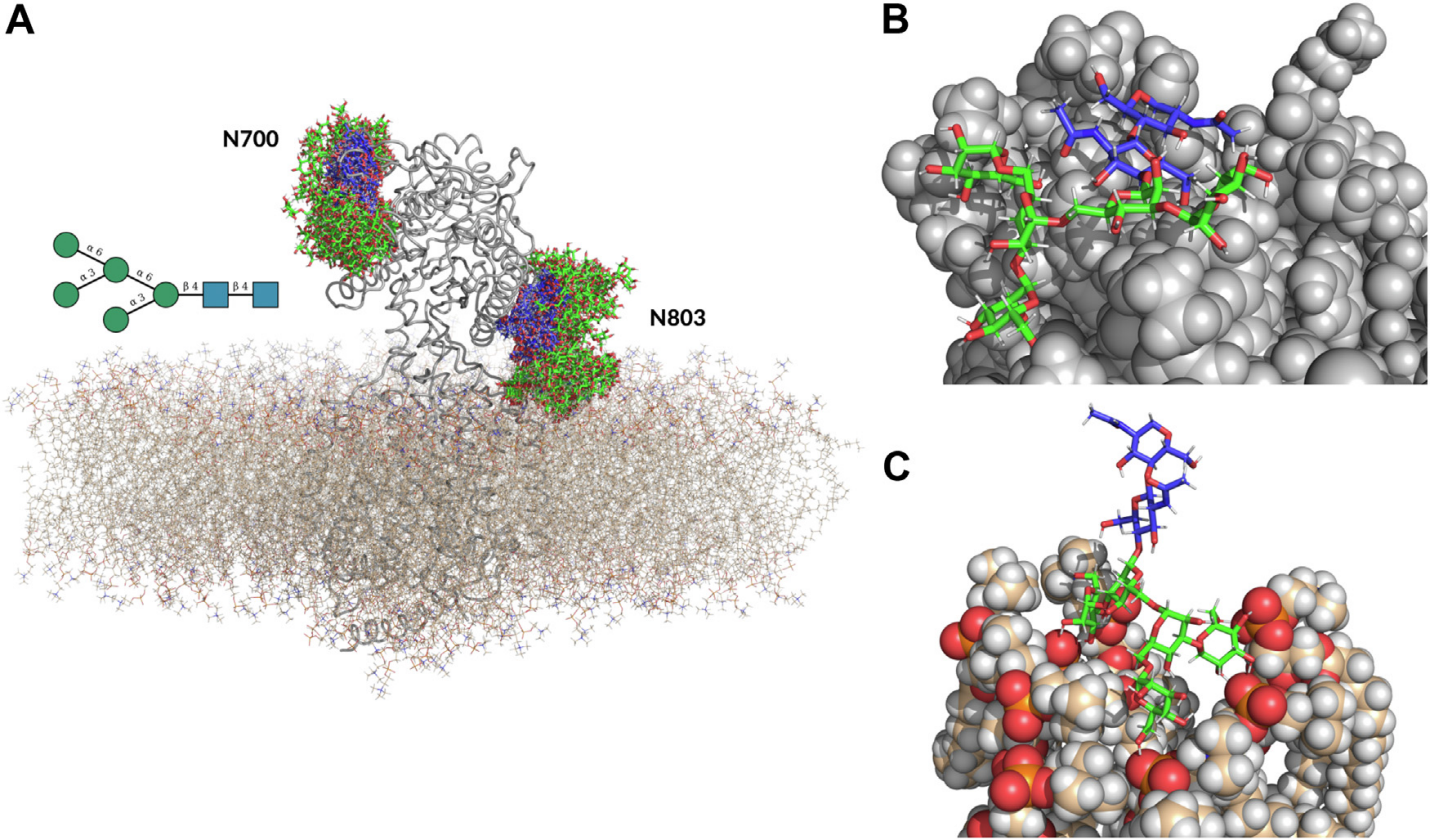
**All-atom molecular dynamics of glycosylated NPC1 demonstrates how the structural landscape can explain the site-specific glycosylation patterns.***A*, graphical representation of NPC1 protein (*grey cartoon*) embedded in a POPC membrane (*tan sticks*). The conformational dynamics of the Man5 oligomannoses at N^700^ and N^803^ is shown by multiple frames taken at 20 ns intervals from the MD trajectory, with the N-glycan structures rendered with sticks with the colours of the C atoms corresponding to the SNFG convention, *i.e.*, green for Man and blue for GlcNAc. *N-*glycans at N^175^, N^327^, and N^766^, as well as water molecules and ions, are not shown for clarity. *B*, snapshot of the Man5 glycan at N^700^ forms interacting with the protein backbone (*grey van der Waals spheres*). This results in the glycan often adopting an inaccessible “folded-back” structure. *C*, snapshot of the terminal residues of Man5 of N^803^ extensively interacting with the phosphorylcholine headgroup of the POPC lipids (van der Waals spheres coloured by atom type). Molecular rendering with PyMol (Schrödinger and DeLano 2020).

During the course of the MD simulations, we observed that the structures of the oligomannose N-glycans at N^175^, N^327^, and N^766^ were all oriented away from the lipid bilayer, facing the bulk water, and thus readily available for functionalization. Meanwhile, the conformational dynamics of the N-glycans at N^700^ and N^803^ show that these oligomannosidic glycans may be inaccessible to glycan-processing enzymes because of steric occlusion, extensive interactions with the protein surface, and the vicinity of the site to the bilayer (Fig. 3*A*). More specifically, the Man_5_GlcNAc_2_ at N^700^ shows extensive interactions with the NPC1, folding-back onto the protein surface, and thus adopting conformations that make the N-glycan inaccessible (Fig. 3*B*). On the other hand, the N-glycan at N^803^ is located between the protein and the lipid bilayer, with the highest populated conformers pointing towards the bilayer, with the terminal mannose residues and the phosphoryl headgroups of the POPC membrane (Fig. 3*C*). We would like to note that as the NPC1 structure was determined by AlphaFold as a recombinant protein mode, it may be possible that in the real-case scenario the α-helix where the N^803^ site is located could sit even closer to the lipid bilayer, making the N-glycan even less accessible.

### Insect-Produced Hemagglutinins are Modified With Difucose and Phosphorylcholine Motifs

**Fig. 4:**
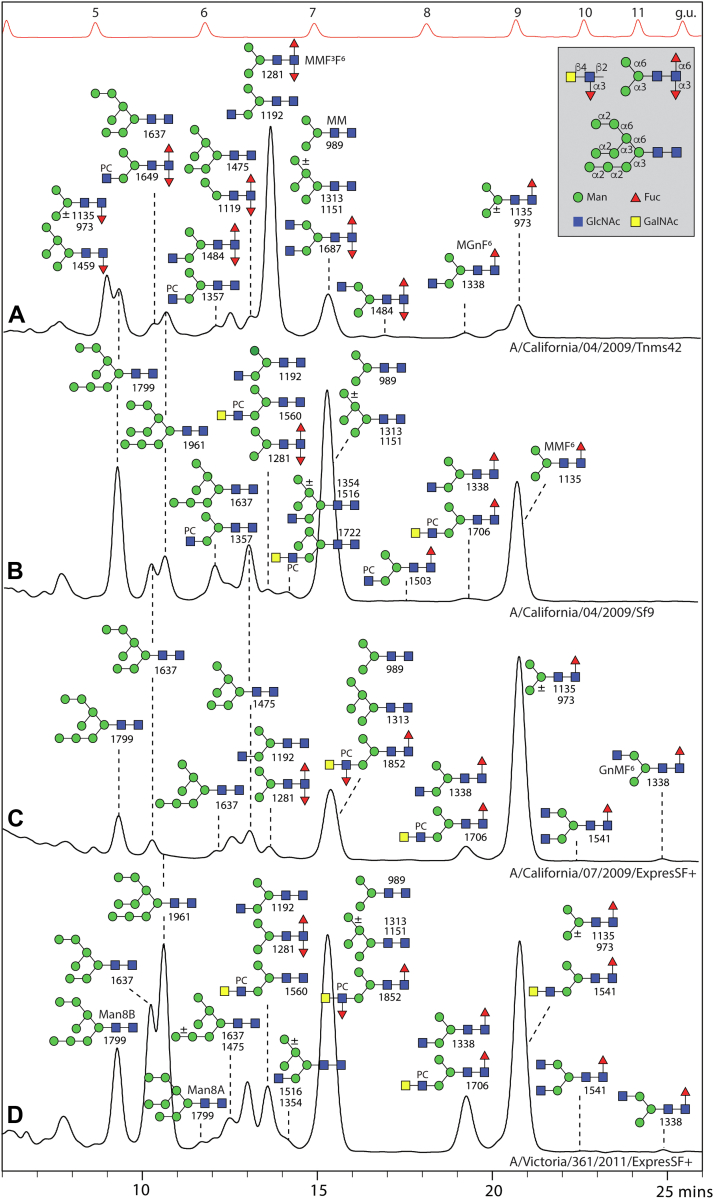
**Analysis of N-glycans of insect-derived recombinant hemagglutinins.** RP-amide-HPLC chromatograms of pyridylaminated N-glycans, released from different hemagglutinins (A and B, A/California/04/2009; C, A/California/07/2009; D, A/Victoria/361/2011) expressed in different insect cell lines (either *T. ni* Tnms42, *S. frugiperda* Sf9 or *S. frugiperda* ExpresSF+), annotated with the structures concluded from co-elution, MALDI-TOF MS and MS/MS. The annotated *m/z* values are for protonated forms detected as [M+H]^+^ in the positive ion mode. Structures are shown in the order of abundance in the relevant fraction (most abundant shown uppermost) according to the Symbol Nomenclature for Glycans, whereby PC indicates phosphorylcholine (see key for linkage information). The elution positions of the isomaltose standards are indicated (5–12 glucose units; g.u.). For Western blot data for recombinant hemagglutinins and MS or MS/MS of individual glycan HPLC fractions refer to Supplemental Figs. S8–S10.

We performed glycan analysis of different influenza hemagglutinins expressed in different insect cell lines (for a summary, refer to Supplemental Table S3): specifically one form (A/California/04/2009 isolated during the swine flu outbreak in 2009 (49)) expressed either in a derivative of *Trichoplusia ni* cells (‘homemade’) or in *S. frugiperda* Sf9 cells (commercial) or two contrasting forms (A/California/07/2009 and A/Victoria/361/2011) produced in the proprietary *S. frugiperda* ExpresSF+ cell line, whereby the sequence of the A/California/07/2009 hemagglutinin (six potential N-glycosylation NXS/T sites; H1N1) is 99% identical to that from A/California/04/2009 (one Ala/Thr difference as verified by tryptic peptide mapping; Supplemental Figs. S8) and 43% to that from A/Victoria/361/2011 (twelve potential N-glycosylation NXS/T sites; H3N2). Glycans released after recombinant PNGase A treatment of tryptic peptides derived from these proteins were labeled with 2-aminopyridine and subject to HPLC and MALDI-TOF-MS/MS (Fig. 4 and Supplemental Figs. S9 and S10). Isomeric structures were distinguished based on the MS/MS spectra as well as comparison to previously published retention times (9).

The two California/04 proteins were contrastingly glycosylated in the two cell lines: whereas for the form produced in *T. ni* cells (50) the paucimannosidic MMF^3^F^6^ and other difucosylated structures dominated (Fig. 4*A* and Supplementary Fig. S10, *O*, *Q*–*T*; with hallmark *m/z* 592 Y1 fragment), expression in Sf9 cells resulted in modification rather with oligomannosidic and simple paucimannosidic glycans, but still some MMF^3^F^6^ was detected (Fig. 4*B*). In both cases, traces of phosphorylcholine-carrying hybrid glycans were found within the pool of released glycans (for MS/MS, see Supplemental Fig. S10, *H*, *I*, *K* and *M*, with hallmark *m/z* 369 and 572 B fragments).

**Fig. 5:**
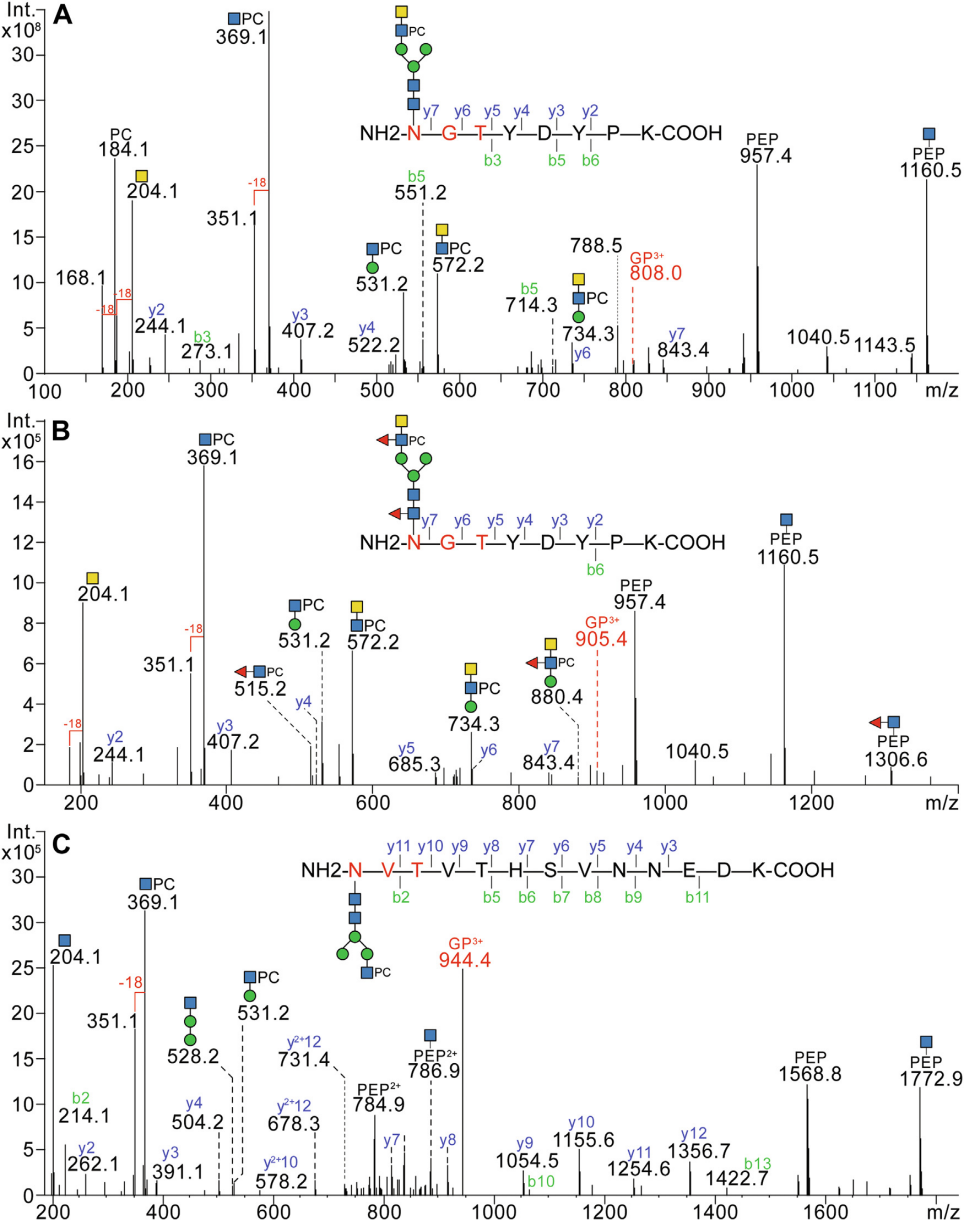
**Glycoproteomic analysis of hemagglutinin N-glycopeptides carrying phosphorylcholine residues**. Sf9-derived A/California/04/2009 hemagglutinin was subject to tryptic digestion and LC-MS/MS analysis. Example spectra are shown for triply-charged glycopeptide precursor ions corresponding to the peptide sequence NGTYDYPK with HexNAc_4_Hex_3_PC_1_ (A; *m/z* 808.0) or HexNAc_4_Hex_3_Fuc_2_PC_1_ (B; *m/z* 905.4) glycans or to NVTVTHSVNNEDK with an HexNAc_3_Hex_3_PC_1_ glycan (C; *m/z* 944.4). Those PC-containing glycopeptides (*A*–*C*) exhibited strong PC-related oxonium ions at *m/z* 184.1 (PC), 351.1 (HexNAc_1_PC_1_-H_2_O), 369.1 (HexNAc_1_PC_1_), 531.2 (HexNAc_1_Hex_1_PC_1_), 572.2 (HexNAc_2_PC_1_), and 734.3 (HexNAc_2_Hex_1_PC_1_). The presence of Y1-peptide ions (Pep+HexNAc) and HexNAc oxoniums (*m/z* 204.1) further validate the glycopeptide identification. For example spectra of oligo- and paucimannosidic glycopeptides, refer to Supplemental Fig. S12.

A glycoproteomic analysis of the Sf9-produced form of California/04 hemagglutinin resulted in the identification of four (N^40^, N^293^, N^304,^ and N^498^) out of six potential glycosylation sites, all with a range of glycan modifications. Based on the occurrence of typical glycan-derived oxonium ions (m/z 204 and 366; HexNAc_1_Hex_0-1_), Y-series fragments (peptide+HexNAc) and y/b peptide fragments, oligomannosidic glycans (Hex_5-9_HexNAc_2_) and paucimannosidic or hybrid glycans (*i.e.*, Hex_1-4_HexNAc_2-3_Fuc_0-1_) could be identified on all sites (Supplemental Figs. S11–S13 and Supplemental Data File). As judged by use of a customized mass list and the occurrence of intense *m/z* 184 (PC_1_), 351 (HexNAc_1_PC_1_-H_2_O), 369 (HexNAc_1_PC_1_) and 572 (HexNAc_2_PC_1_) fragment ions, as found for released phosphorylcholine-modified glycans, in addition to lower intensity HexNAc_2_Hex_1_Fuc_0-1_PC_1_ ions at *m/z* 734 and 880, the presence of a range of zwitterionic N-glycans at all sites was demonstrated (Fig. 5 and Supplemental Data File).

For the two hemagglutinins expressed in the ExpresSF+ cell line, the glycan profiles contrasted in terms of the major glycans. The California/07 form was especially rich in the mono-fucosylated paucimannosidic MMF (6) structure (Fig. 4*C*, Supplemental Figs. S9, and S10), while the Victoria/361 also carried many oligomannosidic glycans (Fig. 4*D*). Both proteins carried traces of difucosylated MMF^3^F^6^ (Supplemental Fig. S10*P*, *m/z* 592 Y1 fragment), LacdiNAc (Supplemental Fig. S10*E*, *m/z* 407 B2 fragment) and phosphorylcholine-modified N-glycans (Supplemental Fig. S10, *J* and *L, m/z* 369 and 572 B fragments), including one structure with a LacdiNAc antennae co-substituted with a “Lewis-X-like” fucose and a phosphorylcholine residue (Supplemental Fig. S10*N, m/z* 718 B2 fragment). All these variants have been previously found in *T. ni* (9) as well as in the cellular glycoprofiles (Figs. 1 and 2). Thus, combining both glycomic and glycoproteomic approaches, our study indicates that *S. frugiperda* and *T. ni* cells have overlapping glycorepertoires, whereby the anti-horseradish peroxidase and C-reactive protein staining upon Western blotting (Supplemental Figs. S8 and S9) are a further indication of the respective occurrence core α1,3-fucose and phosphorylcholine modifications (3, 9, 51).

### Insect-Produced Spike Protein is Modified with Difucose, Sulfate, and Phosphorylcholine

**Fig. 6:**
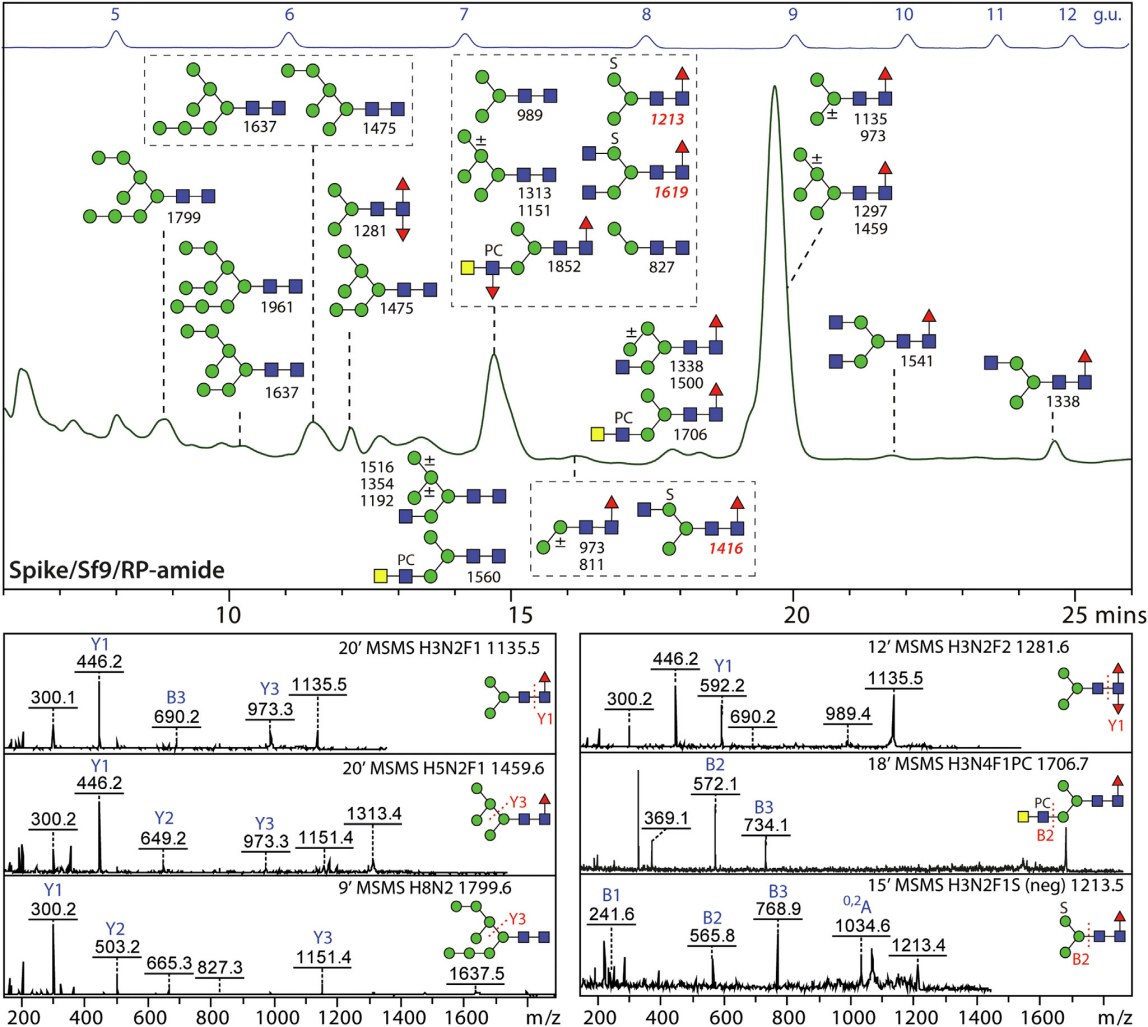
**Analysis of N-glycans of insect-derived SARS-CoV-2 Spike protein.** The RP-amide-HPLC chromatogram of N-glycans (pyridylaminated), released from Spike expressed in Sf9 cells, annotated with the structures concluded from co-elution, MALDI-TOF MS and MS/MS. The annotated *m/z* values are for [M+H]^+^ in the positive ion mode (*black*) or [M-H]^-^ in the negative ion mode (*red*). Structures are shown in the order of abundance in the relevant fraction (most abundant shown uppermost or on the top left of the relevant box) according to the Symbol Nomenclature for Glycans, whereby PC and S indicate phosphorylcholine and sulfate. The elution positions of the isomaltose standards are indicated (5–12 glucose units; g.u.) and aided isomeric identifications. Selected MS/MS spectra of structures present in different HPLC fractions are also shown, labeled with the retention time in minutes. Structures with core difucosylation, phosphorylcholine, and sulfation of mannose are as in other complete lepidopteran N-glycomes but do not occur on mammalian glycoproteins. For Western blot data for the Spike protein and MS of the individual glycan HPLC fractions refer to Supplemental Fig. S14.

The full-length Spike protein in Nuvaxovid (22 potential N-glycosylation sites) is also expressed in an *S. frugiperda* cell line and the glycan analysis was performed similarly as for the hemagglutinins; a previous study only categorized the glycans into oligomannosidic and complex/paucimannosidic without presenting further details (15). In our study, the overall N-glycan profile was not dissimilar to the California/07 hemagglutinin expressed in ExpresSF+ cells (Fig. 6 and Supplemental Fig. S14). The major oligosaccharide was MMF^6^ (*m/z* 446 Y1 fragment), but typical oligomannosidic glycans as well as traces of difucosylated MMF^3^ F^6^ (m/z 592 Y1 fragment) and three phosphorylcholine-modified structures (*m/z* 572 B2 fragment) were present; furthermore, some sulfated N-glycans were also detected in negative mode with the characteristic retention time and MS/MS pattern (*m/z* 565 B2 and m/z 1034 cross-ring ^0,2^A fragments) typical for such structures from insects (9). Staining upon Western blotting with anti-horseradish peroxidase, *A. aurantia* lectin and CRP independently verified the presence of both core α1,3- and α1,6-fucose as well as of phosphorylcholine modifications (Supplemental Fig. S14).

**Fig. 7:**
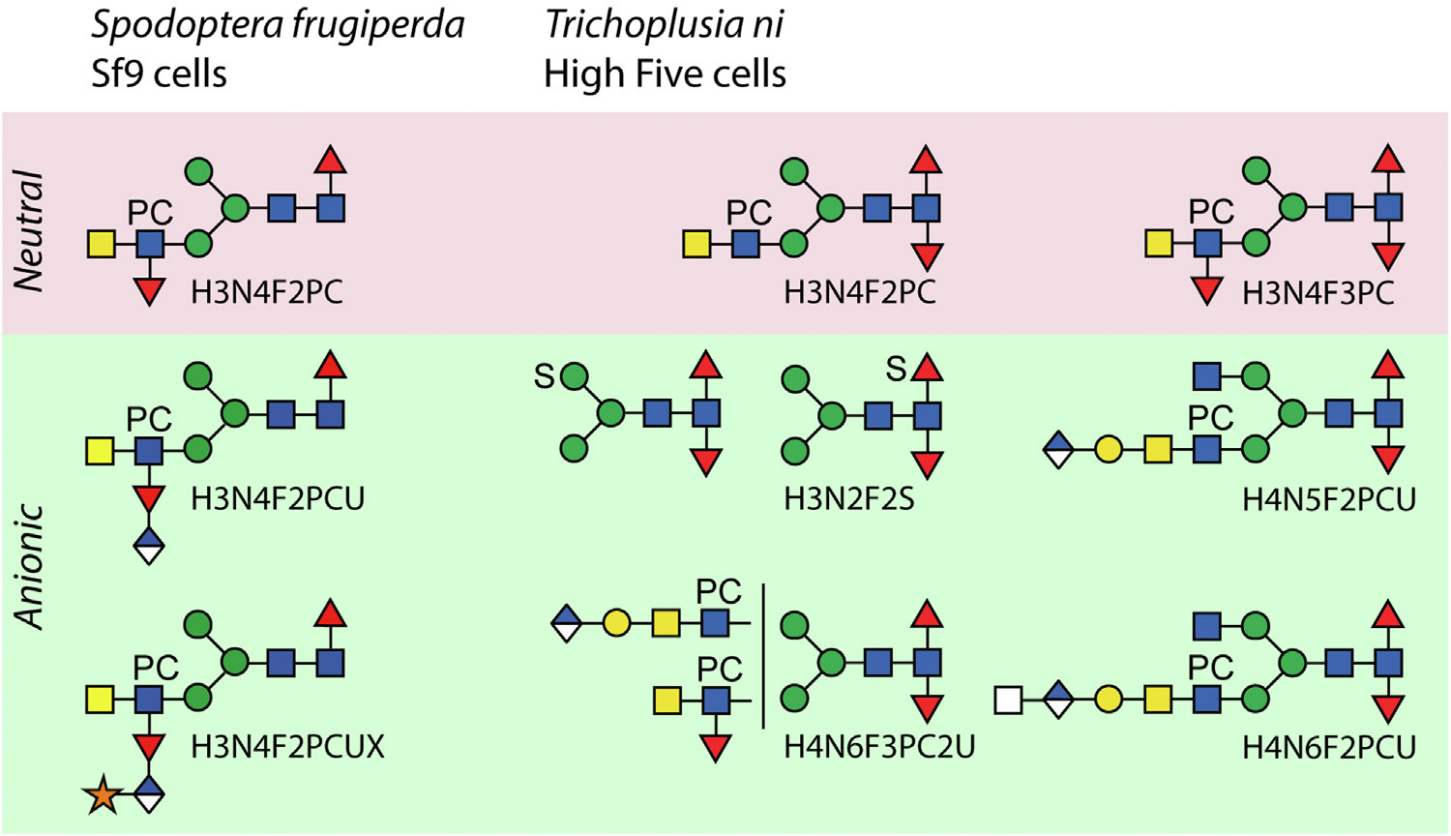
**Species-specific N-glycans from Sf9 and High Five cells.** Examples of proposed glycan structures from the neutral and anionic pools specific to either cell line are shown based on MS/MS data before and after chemical or enzymatic treatments. While the modification of the antennal GlcNAc by a phosphorylcholine is shared, the isomers of Hex_3_HexNAc_2_Fuc_2_PC_1_ differ in terms of position of the fucose residues, whereby difucosylation is more pronounced in High Five cells. The hexuronic acids have quite different locations: on the antennal fucose in Sf9 cells or attached to galactosylated LacdiNAc units in High Five cells. The pentose modification is present only in Sf9 cells. Sulfation of core difucosylated glycans occurs in two positions (mannose or fucose) in High Five cells.

## Discussion

The remarkable degree of glycan diversity in insect cells has only recently become obvious. Here we have examined the endogenous glycoproteome and N-glycome of two popular lepidopteran cell lines as well as recombinant viral glycoproteins produced in the same or derivative cell lines. Despite many previous studies on High Five and Sf9 cells, our data offer surprises, and we demonstrate new structures including species-specific complex N-glycan variations (Fig. 1). For High Five cells, core difucosylated glycans with extended antennae were not found in our previous study (9), but this is probably due to using PNGase Ar with its higher specific activity and broader specificity than native PNGase A and so is another reason to favor the more recently available enzyme for release of difucosylated glycans from invertebrate samples (52). On the other hand, the lower amount of cell weight employed accounts for the underrepresentation of some low-abundance sulfated structures in the current analysis. As compared to other insects, the α1,3-fucosylation of antennal GlcNAc residues is also known from honeybee, mosquito, and other moth glycomes (8, 9, 11, 53, 54). The terminal HexNAc-HexA-Hex motif predicted for two High Five glycans is reminiscent of the repeat unit found on an O-glycoprotein expressed in the High Five cell line (10); in sulfated form, this was also observed in honeybee and mosquito N-glycomes (8, 11).

Unexpectedly, despite *T. ni* and *S. frugiperda* being both members of the Noctuidae family, the most complex structures in High Five cells are not shared with the Sf9 glycome (see Figure 7 and Supplemental Fig. S6). Indeed, rather a new motif with glucuronic acid and xylose modification of fucose was revealed by our analyses of Sf9 cells. Initial hints for these glycans came from compositions present amongst the glycan masses reconstructed from the glycoproteomic data (Fig. 2). Subsequent assessment of the masses present amongst the released and fractionated N-glycans, complemented by data upon hydrofluoric acid treatment, verified this novel motif in the Sf9 glycome (Fig. 1). This demonstrates that the two different approaches result in compatible data. Although the exact linkages of the glucuronic acid to fucose and xylose residues to glucuronic acid are unknown, there are precedents for modifications with these compositions. O-linked fucose in *Drosophila* has been previously concluded to be β1,4-glucuronylated (55, 56) and antennal fucose residues carrying 1,4-linked glucuronic acid are known from mollusc N-glycans (42, 57) or plant rhamnogalacturonan II side chain A (58), whereas mammalian matriglycan, a modification of O-glycans attached to dystroglycan, is a chain based on β1,3-linked glucuronic acid and α1,3-linked xylose (43). Overall, it is the anionic subpools of the N-glycomes of both cell lines that showed the most novelty; based on HPLC peak intensity, these subpools account for 5% of the total N-glycome. Thus, beyond the well-established occurrence of paucimannosidic and simpler LacdiNAc-modified glycans, the detected hybrid and biantennary glucuronylated structures have not been previously described for any organism; either their low abundance or protein-specific factors result in not all these glycans being detected on the recombinant viral proteins or in the cellular glycoproteome.

Among the proteins identified in the glycoproteomic study, the NPC1 showed site-specific glycosylation patterns in both cell lines, whereby some sites were modified with processed glycans and others retained oligomannosidic structures. The molecular modeling of the fully glycosylated NPC1 structure (Fig. 3) highlights that the determined glycosylation patterns can be reconciled with the location of the N-glycosylation sites relative to the bilayer and with the complementarity of the N-glycans to the surrounding protein landscape, in agreement with earlier case studies (59, 60, 61) indicating that exposed glycosylation sites will be more highly processed as compared to ‘hidden’ ones.

Various studies have reported the glycosylation of recombinant viral glycoproteins expressed in insect cells using the baculovirus system, with probably the first report being over 30 years ago, showing the presence of simple paucimannosidic and oligomannosidic N-glycans on an avian influenza hemagglutinin (62). In the intervening years, many either complete glycomes of recombinant hemagglutinins or glycoproteomic analyses of hemagglutinins or Spike have been published, either based on the use of the same protein sequences or the same cell lines as examined here. Most studies on expression in *T. ni* cells were performed on High Five cells, and most of these concur that at least some portion of the N-glycans were difucosylated (50, 63, 64, 65). However, in two glycoproteomic studies, searches of the data for “peptide+HexNAc+2Fuc” were apparently not performed, and so only Hex_2-5_HexNAc_2_Fuc_0-1_ structures were reported on either a hemagglutinin or Spike (66, 67); the possibility that there may be anionic or zwitterionic glycans was not explored. In another study, a different *T. ni* cell line reported the glycosylation of an H5N1 hemagglutinin in Tn-NVN cells (68), which apparently endogenously lack core α1,3-fucose (69). In our study, the Tnms42 cell line was the “virus-free” derivative of High Five used as the expression host for one of the hemagglutinins (70); thus, in the context of the whole cell glycome analyses of the parental line, a high degree of core α1,3-fucosylation and a low level of phosphorylcholine-modified structures is not unexpected.

When considering expression in *S. frugiperda* cells, seemingly two different cell lines (the “classical” Sf9 and the proprietary ExpresSF+) have been used for expression of hemagglutinin or Spike. Some studies only report categories of glycans (“paucimannosidic,” “oligomannosidic,” “complex”) and not specific structures (15, 71), but others are more detailed. Generally, no difucosylation was previously reported on viral glycoproteins expressed in either Sf9 or ExpresSF+ cells, rather a preponderance of the paucimannosidic (with and without one fucose) and oligomannosidic glycans (50, 63, 72, 73). In a glycoproteomic study on SARS-CoV-2 Spike expressed in ExpresSF+ cells, a certain degree of difucosylation was detected, with up to 5% on some sites (74); such a low level is compatible with our own data on Sf9-produced Spike. On the other hand, we are the first to report any anionic and zwitterionic structures on Sf9-produced recombinant glycoproteins. In enzymatic assays, Sf9 cells possessed no detectable core α1,3-fucosyltransferase activity under the employed conditions (75), whereas whole glycome analyses indicated that the parental cell line, Sf21, contains difucosylated N-glycans (2); overall, we conclude that *S. frugiperda* cell lines can express at least four non-mammalian N-glycan epitopes: core difucose, phosphorylcholine, hexuronylation of antennal fucose, and sulfation of mannose.

Another comparison that can be made is that of older and the current studies on recombinant forms of the same proteins: indeed, there have been a number of reports regarding glycans on A/California/07/2009 or the 99% identical A/California/04/2009 hemagglutinins (73). Comparing these data indicates that we identify four (N^40^, N^293^, N^304^ and N^498^; numbering including the signal sequence) of six sites but find at these more structures with our search approach, including phosphorylcholine-modified glycans. One of the sites we did not identify is on a long tryptic glycopeptide (N^104^); ideally, a second protease should be used to detect this site. Unfortunately, for SARS-CoV-2 Spike expressed in Sf9 or High Five cells, often either only categories of glycans were reported or the search criteria apparently excluded difucosylated, sulfated, or phosphorylcholine-modified glycans. In two studies, cell-dependent variations in the levels of difucosylation (estimated five or 44%) were reported for Spike expressed in either ExpresSF+ cells or High Five cells (64, 74), but only low levels (≥1%) of glycans with four HexNAc residues were reported. For a commercially-purchased Spike expressed in “baculovirus-insect cells” (*i.e.*, species not defined), the level of difucosylation (also 40–45%) is suggestive that actually HighFive cells were used (76). In contrast, in mammalian HEK293 cells, there is a high degree of complex glycosylation on the 22 N-glycan sites (64, 77). As paucimannosidic forms in insect cells also depend on Golgi processing, the dominance of Man_3_GlcNAc_2_Fuc_1_ shown here for the Sf9-derived material (Fig. 6) shows that the glycans on the surface of the Spike protein are accessible to various secretory pathway enzymes regardless of the expression system.

With our data, it is obvious that non-mammalian epitopes are being overlooked and the repercussions are unclear, considering that difucosylated motifs are known ligands for IgE of honeybee venom allergic patients or the IgG of *Trichuris*-infected pigs (78, 79); phosphorylcholine-modified N-glycans on the nematode ES-62 protein are immunomodulatory (80), but zwitterionic glycans are also recognized by sera of nematode-infected animals (79, 81); indeed, some of the glycan fractions bound by IgG from *Trichuris*-infected pigs contain the same PC/Fuc-disubstituted LacdiNAc motif as found in both Sf9 and High Five cells. Furthermore, glycovariations between sources of recombinant proteins will affect which proteins of the human innate immune system can bind, whereby mannose-binding lectin bound to Spike can activate the complement pathway (82), whereas phosphorylcholine-modified glycans may result in abortive complexes of CRP with early complement factors (83).

While reengineering insect cells to abolish fucosylation has been done (50), the enzymes required for sulfation or phosphorylcholinylation are unknown; typical modifications of the glycosylation pathway in insect cells over the past 30 years have centered on abolishing N-glycan-modifying hexosaminidase activities and/or promoting generation of mammalian-type antennae. Potentially, these approaches lead to outcompeting the insect-type complex modifications, but this remains to be proven; indeed, knock-out of the FDL hexosaminidase in *Drosophila* S2 cells led to an apparent increase in glucuronylated structures (84). Certainly, we recommend that those studying glycosylation of recombinant pharmaceutical products produced in insect cells first perform an unbiased whole glycosylation analysis. Although done in some cases (63), permethylation should be avoided for initial screening as phosphorylcholine is apparently lost, whereas for sulfation, special extraction procedures are required (85). Finally, as performed here for the A/California/04/2009 hemagglutinin, adaptation of glycan libraries used for database analysis or searching for zwitterionic fragment ions is recommended so as to include all possible insect-type modifications; the comparison of various programs employed on the hemagglutinin glycopeptide data suggested that FragPipe was the only one able, under the employed settings, to identify the phosphorylcholine-modified N-glycans.

In conclusion, our glycomic data indicate that lepidopteran cells express a range of zwitterionic, anionic, and difucosylated N-linked oligosaccharides not just on their own endogenous glycoproteins but that these motifs are present on recombinant vaccines and vary between the host cell lines (Figure 7, Supplemental Tables S1 and S2). While many structures are shared between High Five and Sf9 cells, the glucuronylated N-glycans display species-specific variations. The data fit well with the concept that cells have a “glycotype” in terms of glycan processing but also show a highly non-human “glycopotential.” Thus, recombinant proteins produced in insect cell lines are potentially immunogenic and/or immunomodulatory, which may affect their use as therapeutic agents or as tools in academic research. Thus, not only should the glycomic workflow used to analyze recombinant glycoproteins from insect cells consider the presence of such structures, but strategies to modulate their expression should be developed.

## Data Availability

The mass spectrometry proteomics and glycoproteomics data have been deposited to the ProteomeXchange Consortium *via* the PRIDE partner repository (86) with the dataset identifiers PXD049172 and PXD051441 and can be visualised using MS-Viewer (87) (keys dvrnilejop, auwnsm3soh, mqo4pvdewk, vmtudwf1qj, l7quytauaq, c7jbc0kkjx, lbmfjrr6h3 and fe4uvbejeu).

The MALDI-TOF MS/MS data is available *via* Glycopost (88): https://glycopost.glycosmos.org/entry/GPST000369.

## Supplemental data

This article contains supplemental data. The Supplement contains further information on the glycomics and glycoproteomics experiments as well as Supplemental Figs. S1–S14, Supplemental Tables S1–S3 and a Supplemental Data xlsx file (8, 9, 10, 11, 13, 19, 24, 79, 87).

## Conflict of interest

The authors declare that they have no conflicts of interest with the contents of this article.

## Supplemental Data

Supplement_Yan_Vanbeselaere
