# Supplementary material for: Glycoproteomic and Single-Protein Glycomic Analyses Reveal Zwitterionic N-Glycans on Natural and Recombinant Proteins Derived From Insect Cells
Source: Mol Cell Proteomics. 2025 May 5;24(6):100981. doi: 10.1016/j.mcpro.2025.100981 (PMC12166434; doi:10.1016/j.mcpro.2025.100981)
Supplement: Supplement_Yan_Vanbeselaere [file mmc1.pdf]

Glycoproteomic and single-protein glycomic analyses reveal zwitterionic N-glycans on natural and recombinant proteins derived from insect cells

Shi Yan (闫石) [1,2], Jorick Vanbeselaere[1], Callum Ives [3], David Stenitzer[1], Lena Nuschy[1], Florian Wöls[1], Katharina Paschinger[1], Elisa Fadda [3,4], Johannes Stadlmann[1], and Iain B. H. Wilson[1,*]

[1] Institut für Biochemie, Universität für Bodenkultur, 1190 Wien, Austria
[2] Institut für Parasitologie, Veterinärmedizinische Universität, 1210 Wien, Austria
[3] Department of Chemistry, Maynooth University, Maynooth, Ireland
[4] School of Biological Sciences, University of Southampton, SO17 1BJ, United Kingdom
* E-Mail: iain.wilson@boku.ac.at

Supplementary Information

Further information regarding the glycoproteomic analyses

Protein And Peptide Identification for High Five and Sf9 cells:

- **All peptide sequences assigned:** Two tables of all semi-tryptic glycopeptides, including their modifications and search engine score values, are provided in the Supplementary Information (i.e., sheets Sf9_msConvert_Comet_semiT_NST-HexNAc and Hi5_msConvert_Comet_semiT_NST-HexNAc in Supplementary_Data_File.xlsx)
- **Precursor charge and mass/charge:** These parameters are listed for each peptide assignment in the same tables.
- **All modifications observed:** Two tables of all semi-tryptic glycopeptides, including their modifications and search engine score values, are provided in the Supplementary Information (i.e., sheets Sf9_msConvert_Comet_semiT_NST-HexNAc and Hi5_msConvert_Comet_semiT_NST-HexNAc in Supplementary_Data_File.xlsx)
- **Number of matched and unmatched masses not applicable**
- **Score(s):** Two tables of all semi-tryptic glycopeptides, including their modifications and search engine score values, are provided in the Supplementary Information (i.e., sheets Sf9_msConvert_Comet_semiT_NST-HexNAc and Hi5_msConvert_Comet_semiT_NST-HexNAc in Supplementary_Data_File.xlsx)
- **Protein accession number and sequence database or spectral library source.** Species specific proteome sequence data-based (i.e. UP000829999 for Sf9, UP000322000 for Hi5 cells) were obtained from uniprot.org
- **Protein sequence % coverage:** not applicable.
- **Proteins identified on basis of a single unique peptide:** not applicable.
- **Ability to view annotated mass spectra:** For visualization of the Comet search results as presented in the Supplementary Data File with e.g. “MS-Viewer” (ProteinProspector; https://msviewer.ucsf.edu/mshome.htm) we provide .mgf files of all intermediate MS/MS data processing steps in PXD049172. More specifically:
  - All .raw files msConvert converted into mgf-files with identical file name (but of file-type .mgf; e.g., 01072022_Sf9_HILIC25.raw -> 01072022_Sf9_HILIC25.mgf).
  - Charge-deconvoluted and deisotoped MS/MS data as .mgf files with the file name tag “DD” (e.g., 01072022_Sf9_HILIC25_DD.mgf).

- SugarQbits processed and precursor-mass corrected MS/MS data are provided as .mgf files with the file name tag “Deglyco” (e.g., 01072022_Sf9_HILIC25_DD_Deglyco.mgf).
- MS/MS data stripped from glycan-derived oxonium ion signals are provided in form of .mgf files with the name tag “RepX” (e.g., 01072022_Sf9_HILIC25_DD_Deglyco_RepX.mgf). These files were the input for subsequent Comet searches and gave rise to the results reported in this manuscript.

Please note that we also provide .mgf files with the file name tag “REFINE”. These .mgf files have been generated using PEAKS X Pro Studio 10.6 and are provided in searchgui_out.zip file format; they can be only visualized in the SearchGUI/PeptideShaker environment (https://doi.org/10.1038/nbt.3109). Furthermore, the MS/MS spectral data can be visualized by MSViewer (https://doi.org/10.1074/mcp.O113.037200):

Sample	Key	Description
Hi5	dvrnilejop	msConvert extracted MS/MS data of raw files in .mgf format<br>Hi5_rawMGFs_merge.mgf:<br>https://msviewer.ucsf.edu/prospector/cgi-bin/mssearch.cgi?report_title=MS-Viewer&search_key=dvrnilejop&search_name=msviewer
Hi5	auwnsm3soh	deisotoped & charge-deconvoluted raw MS/MS data; with Oxonium ions and glycan derived fragment ions removed<br>Hi5_DD_RepX_merge.mgf:<br>https://msviewer.ucsf.edu/prospector/cgi-bin/mssearch.cgi?report_title=MS-Viewer&search_key=auwnsm3soh&search_name=msviewer
Hi5	mqo4pvdewk	SugarQBits processed, precursor mass corrected MS/MS data (from deisotoped & charge-deconvoluted MS/MS data; with Oxonium ions and glycan derived fragment ions removed)<br>Hi5_DD_RepX_Deglyco_merge.mgf:<br>https://msviewer.ucsf.edu/prospector/cgi-bin/mssearch.cgi?report_title=MS-Viewer&search_key=mqo4pvdewk&search_name=msviewer
Sf9	vmtudwf1qj	msConvert extracted MS/MS data of raw files in .mgf format<br>Sf9_rawMGFs_merge.mgf:<br>https://msviewer.ucsf.edu/prospector/cgi-bin/mssearch.cgi?report_title=MS-Viewer&search_key=vmtudwf1qj&search_name=msviewer
Sf9	l7quytauaq	deisotoped & charge-deconvoluted raw MS/MS data<br>Sf9_DD_merge.mgf:<br>https://msviewer.ucsf.edu/prospector/cgi-bin/mssearch.cgi?report_title=MS-Viewer&search_key=l7quytauaq&search_name=msviewer
Sf9	c7jbc0kkjx	SugarQBits processed, precursor mass corrected MS/MS data (from deisotoped & charge-deconvoluted raw MS/MS data)<br>Sf9_DD_Deglyco _merge.mgf:<br>https://msviewer.ucsf.edu/prospector/cgi-bin/mssearch.cgi?report_title=MS-Viewer&search_key=c7jbc0kkjx&search_name=msviewer
Sf9	lbmfjrr6h3	Oxonium ions and glycan derived fragment ions removed (from SugarQBits processed, precursor mass corrected MS/MS data).<br>Sf9_DD_Deglyco_RepX_merge.mgf:<br>https://msviewer.ucsf.edu/prospector/cgi-bin/mssearch.cgi?report_title=MS-Viewer&search_key=lbmfjrr6h3&search_name=msviewer

- **Posttranslational Modifications:** ***The site(s) of modification:*** No site-localization within a given glycopeptide was performed. All site-localization provided by the search-engine are thus “ambiguous”. In our glycoproteomics analyses we did not perform any site localization of N-glycan attachment within the peptide sequences. Instead, we considered the mass of a single HexNAc as a variable modification on any Asn, Ser, and Thr residue. Consequently, in MS-Viewer (and the supplementary data files) glycopeptide sequences are often listed with incorrect glycan position assignments. Noteworthily, MS-viewer enables the fragmentation comparison of positional glycopeptide isomers, which in most cases can unambiguously prove N-glycosylation, even if the search engine indicated otherwise. Glycan masses were determined as the differences calculated from the original precursor masses and the precursor masses of such in silico "deglycosylated" spectra. The accuracy of the glycan masses reported in this study thus strictly depends on the accuracy of the monoisotopic ion assignments of the original precursor ions. ***Ambiguous assignments:*** No site-localization was performed. All site-localization provided by the search-engine are thus “ambiguous”. ***Annotated, mass labeled spectra***: All spectra were submitted to the public repository of the ProteomeXchange Consortium via the PRIDE partner repository with the dataset identifier PXD049172.

Protein And Peptide Identification for a recombinant haemagglutinin:

- **All peptide sequences assigned:** A table of all tryptic peptides and glycopeptides is provided in the Supplementary Information (see Supplementary_Data_File.xlsx)
- **Precursor charge and mass/charge:** These parameters are listed for each peptide assignment in the table.
- **All modifications observed:** The tables of all tryptic peptides and glycopeptides, including their modifications and search engine score values, are provided in the Supplementary Information (see sheets Sf9 A California 04 2009 glyco and Sf9 A California 04 2009 raw in Supplementary_Data_File.xlsx)
- **Score(s):** The tables of peptides and predicted glycopeptides, including search engine score values, are provided in the Supplementary Information (see Supplementary_Data_File.xlsx)
- **Protein accession number and sequence database or spectral library source.** The sequence according to the suppliers' information is based on Uniprot accession C3W5S1, but excludes the signal sequence; the natural C-terminal transmembrane domain is replaced by an octahistidine tag.
- **Protein sequence % coverage:** 74.4% (388 of expected 521 residues).
- **Proteins identified on basis of a single unique peptide:** not applicable.
- **Ability to view annotated mass spectra:** All spectra are uploaded to the PRIDE database. Furthermore, the spectra can be viewed via: https://msviewer.ucsf.edu/cgi-bin/mssearch.cgi?report_title=MS-Viewer&search_key=fe4uvbejeu&search_name=msviewer
- **Posttranslational Modifications:** ***The site(s) of modification:*** Sites of modification were identified by analysis of the MS/MS data using MSfragger; where the exact position was not localised by the software, the single Asn-Xaa-Ser/Thr site within the peptide was assumed to be the site of modification. See the Supplementary Information (Supplementary_Data_File.xlsx), example MS/MS spectra shown in Figure 5 and Supplementary Figure 12 and summary in Supplementary Figure 13.
- ***Annotated, mass labelled spectra***: All spectra were submitted to the public repository of the ProteomeXchange Consortium via the PRIDE partner repository with the dataset identifier PXD051441.

Further information regarding the glycomic analyses

Definition of the level of the glycan structural analysis:

The goal was the N-glycomic analysis of four samples of influenza haemagglutinins and one sample of recombinant SARS-CoV-2 Spike protein, all recombinantly produced in different insect cell lines and to compare these to the overall N-glycomes of two Lepidopteran cell lines (High Five and Sf9). Thereby, either individual glycan-containing HPLC fractions or whole N-glycomes were subject to MALDI-TOF MS and MS/MS.

Search parameters and acceptance criteria:

a. **Peak lists:** As stated in the methods section: typically 1000-4000 shots were summed for MALDI-TOF MS and 5000-20000 for MS/MS. Spectra were processed with the manufacturer's software (Bruker Flexanalysis 3.3.80) using the SNAP algorithm with a signal/noise threshold of 6 for MS (unsmoothed) and 3 for MS/MS (four-times smoothed).
b. **Search engine, database and fixed modifications:** All glycan data were manually interpreted and no search engine or database was employed; the fixed modification is the 2-aminopyridine label at the reducing end ($GlcNAc_1$-PA fragments of *m/z* 300).
c. **Exclusion of known contaminants and threshold:** All glycan data were manually interpreted; only peaks with an MS/MS consistent with a pyridylaminated chitobiose core were included – the 'threshold' for inclusion was an interpretable MS/MS spectrum (at least in terms of composition).
d. **Enzyme specificity:** A description of the PNGase A release method is given in the methods section; the enzyme should remove N-glycans from glycopeptides regardless of the presence of core α1,3-fucose on the reducing-terminal GlcNAc. The lot of PNGase Ar employed for the cell line glycome contained also endoglycosidase H. Exoglycosidase digestion were performed on selected glycan fractions to determine linkages of some glycan motifs.
e. **Isobaric/isomeric assignments:** For isomeric species, differences in RP-HPLC elution and MS/MS were used for the assignment (as described in the text).

Glycan or glycoconjugate identification:

a. **Precursor charge and mass/charge (*m/z*):** All glycans detected were singly-charged. For the positive mode, the *m/z* values are for protonated forms. Depending on the glycan amount or preparation, the relative amounts of the H^+, Na^+ and K^+ adducts varied. Maximally two decimal places used for the *m/z* annotations consistent with the accuracy of MALDI-TOF MS; in the figures and due to space limitations, only one decimal place is presented. Previous data indicate an average +0.03 Da (+ 22 ppm) deviation between the measured and the calculated *m/z* values on the instrument used.
b. **MALDI-TOF MS settings (positive mode):** For Autoflex Speed: Ion Source 1 and 2 were 19.00 and 16.75 kV; Lens, 9.00 kV; Reflector 1 and 2, 21.05 and 9.65 kV; Pulsed Ion Extraction, 160 ns; Matrix Suppression typically up to 700 Da; Detector Gain, typically 2163 V. For Rapiflex: reflector voltage, lens voltage, and gain of 20.8 kV, 11.6 kV, and 1909 V respectively.
c. **MALDI-TOF MS/MS settings (positive mode):** For Autoflex Speed: Ion Source 1 and 2 were 6.00 and 5.35 kV; Lens, 2.90 kV; Reflector 1 and 2, 27.00 and 11.75 kV; Lift 1 and 2, 19.00 and 4.00 kV; Pulsed Ion Extraction, 140 ns; Detector Gain, typically 2260 V when fragmenting; Laser Power Boost typically 50%; not in CID mode; PCIS typically 0.65%. For Rapiflex: reflector voltage, lift voltage, and gain of 23.8 kV, 19 kV, and 2171 V respectively; PCIS 0.3%.
d. **All assignments:** For the glycans present in each pool, see the RP-HPLC chromatograms annotated with structures shown according to the Standard Nomenclature for Glycans.

e. **Modifications observed:** Listed are the *m/z* values for glycans carrying a reducing terminal pyridylamine group as judged by presence of an *m/z* 300 $GlcNAc_1$-PA fragment. As the glycans are otherwise chemically unmodified, $\Delta m/z$ of 80, 132, 146, 162, 165, 176 and 203 correspond to sulphate (detected in negative mode), xylose (pentose), fucose (deoxyhexose), hexose, phosphorylcholine, glucuronic acid (hexuronic acid; detected in positive and negative modes, therefore ruling out the isobaric methylhexose as a possibility) or *N*-acetylhexosamine. There was no indication for the presence of phosphate or sialic acid residues. While pyridylamine is a chemical modification of the N-glycans performed during the glycomic workflow, phosphorylcholine is a natural glycomic modification also found in nematode species and results in appearance of *m/z* 184 fragment ions; phosphorylcholine in mammals occurs as the headgroup of phosphatidylcholine lipids and platelet activating factor, but not as a glycan modification.

f. **Number of assigned masses:** Glycan assignments were not just based on measured mass only, but on the basis of MS/MS corroborated by elution data.

g. **Spectra:** Representative annotated spectra (MS and MS/MS) defining structural elements are given in various figures. In total, over 140 MS or MS/MS spectra reflecting the data for the approximately 90 defined structures are shown in the main text or supplement and 115 mzxml files have been uploaded to Glycopost.

h. **Structural assignments:** As noted in the results section, the typical oligomannosidic structures are assigned based on elution time and fragmentation pattern; it is otherwise assumed that the glycans contain a di- or tri-mannosyl core consistent with typical eukaryotic N-glycan biosynthesis and that there is processing by GlcNAc-transferases and core α1,3/6-fucosyltransferases as in a range of multicellular organisms. Antennal PC-modified fucosylated LacdiNAc motifs have been defined for other lepidopteran N-glycans on the basis of α1,3-fucosidase, β1,4-*N*-acetylgalactosaminidase and hydrofluoric acid treatments (Stanton et al., 2017) as also shown here. The glucuronylated antennae in High Five cells are assumed to be GlcAβ1,3Galβ1,3GalNAcβ1,4GlcNAc, as previously observed in other insect samples (Kurz et al., 2015; Hykollari, et al., 2019); these GlcA residues were shown to be β-glucuronidase-sensitive. The sulphated glycans are observed in negative mode as $[M-H]^-$ ions, but in-source loss results in the observation of $[M+H-80]^+$ ions in positive mode - a property contrasting with phosphorylated glycans which would be observed in both modes. The novel $GlcA_1Xyl_{0-1}$ modifications of antennal fucose residues in Sf9 cells are proposed on the basis that the glycans were observed in both positive and negative modes, that the $Fuc_1GlcA_1Xyl_{0-1}$ element was cleaved by hydrofluoric acid treatment and that insects possess glycoconjugates containing GlcA and Xyl, but are not known to possess GalA or Ara as monosaccharide components.

Summary of glycosidases used in this study

PNGase	*Oryza sativa* PNGase A (recombinant, *Pichia*)
Fucosidase	Bovine α-fucosidase (native)
Glucuronidase	Human β-glucuronidase (recombinant, murine NS0 cells)
Hexosaminidases	Jack bean β-*N*-acetylhexosaminidase (native)<br>*C. elegans* HEX-4 β1,2-*N*-acetylgalactosaminidase (recombinant, *Pichia*)<br>Deep-sea sediment metagenome-derived endo-*N*-acetylgalactosaminidase
Mannosidase	Jack bean α-mannosidase (recombinant, *Pichia*)

Supplementary Figure S1: MALDI-TOF MS analysis of unusual N-glycans from High Five cells. (A) The neutral (black) and anionic (red) subpools of N-glycans from High Five cells were subject to HPLC on an RP-amide column; annotations are based on MS and MS/MS data, also in comparison to studies using the same column (Stanton et al., 2017), with phosphorylcholine-modified glycans highlighted in yellow boxes. (B) Positive mode MALDI-TOF MS/MS of four HPLC-fractionated neutral N-glycans of ca. 2200 Da demonstrating antennal modifications with phosphorylcholine (B ions at *m/z* 369, 572 and 718 corresponding to HexNAc$_{1\text{-}2}$Fuc$_{0\text{-}1}$PC$_1$) similar to those previously found in Lepidoptera and Nematoda (Stanton et al., 2017; Eckmair et al., 2024). (C) Positive and negative mode MALDI-TOF MS of two fractions (10 and 14 mins) from the anionic RP-amide run, together with MS/MS of the major glycan in the 14 min fraction (see insets); note that sulphate residues are lost by ion-source fragmentation in positive ion mode. (D) Positive mode MALDI-TOF MS/MS of four HPLC-fractionated anionic N-glycans demonstrating sulphation on either mannose or fucose or antennal modifications with phosphorylcholine and glucuronic acid; differences of 203 (HexNAc), 368 (HexNAcPC) and 541 (HexNAc$_1$Hex$_1$HexA$_1$) are indicated as are B ions at *m/z* 241 and 225 (i.e., HexS or FucS) or 369, 572, 718 and 910 (i.e., HexNAc$_{1\text{-}2}$Hex$_{0\text{-}1}$HexA$_{0\text{-}1}$Fuc$_{0\text{-}1}$PC$_1$). For further MS/MS refer to ***Figure 1*** in the main text, ***Supplementary Figures S2-S4*** or mzxml files available via Glycopost.

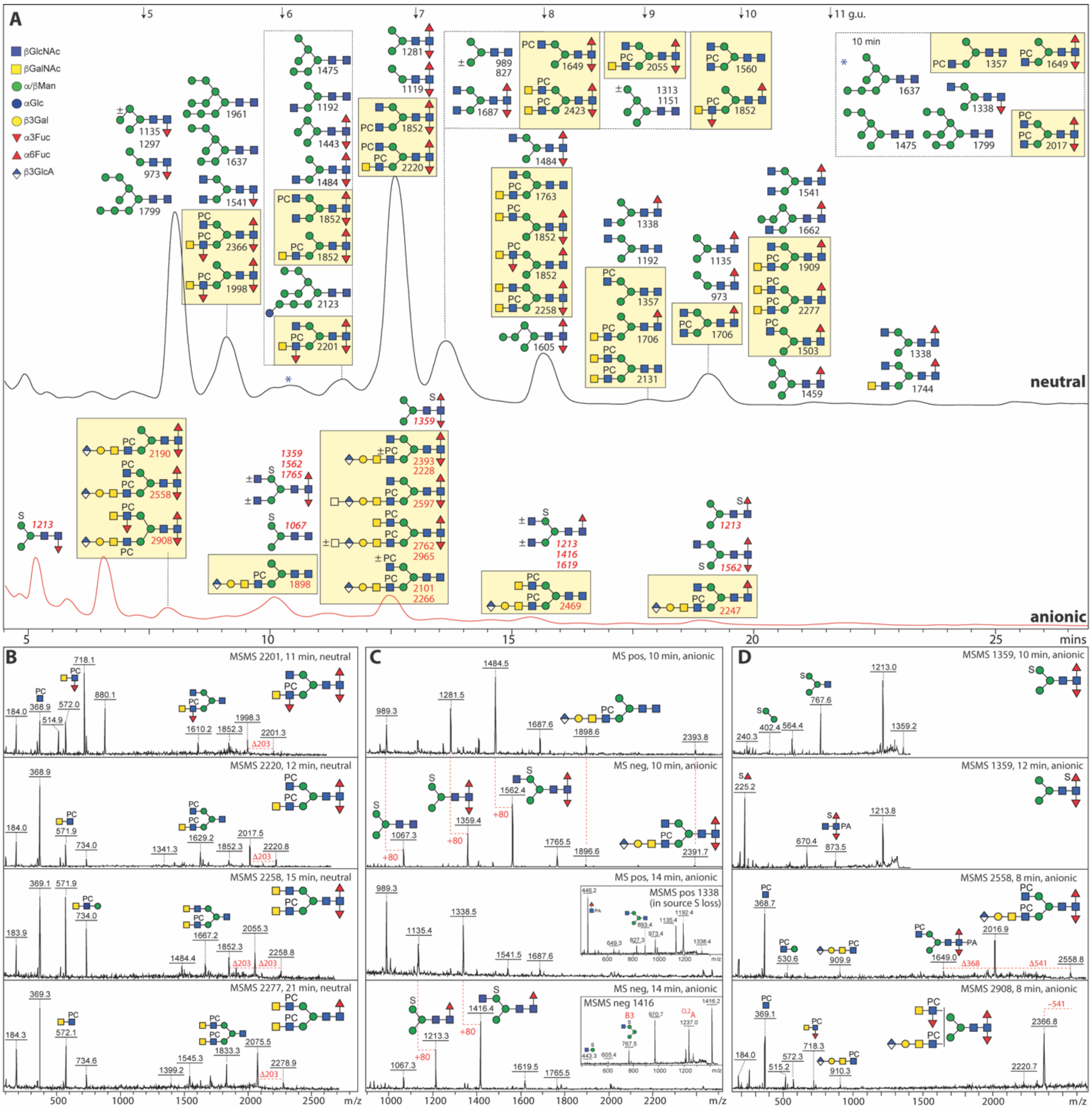

Supplementary Figure S2: Further characterisation of LacdiNAc units in High Five and Sf9 cells. (A and B) MALDI-TOF MS of the neutral Sf9 N-glycan fraction eluting at 13 min (7 g.u.) before and after partial digestion (3 hours) with *C. elegans* HEX-4, which is GalNAc-specific enzyme previously shown to cleave only LacdiNAc rather than chitobiose units, but is less efficient with fucosylated motifs; red dashed lines indicate the loss of 203 Da from either the parent or major B ion, thus demonstrating the position of the antennal fucose residue with respect to the PC moiety. As the GalNAc in LacdiNAc is β1,4-linked, the fucose is α1,3-linked and the C2 of the antennal GlcNAc is substituted by an N-acetyl moiety, it can be deduced that the phosphorylcholine substitutes the C6 position as also found in nematodes. (C and D) MALDI-TOF MS of the neutral High Five N-glycan fraction eluting at 15-16 min (8 g.u.) before and after partial digestion with the GalNAc-specific *C. elegans* HEX-4; a number of glycans lost one or two HexNAc residues, indicative of the sub-terminal position of the PC moiety and confirms the presence of terminal GalNAc. Note that the *m/z* 1852 in the untreated fraction is predominantly a core difucosylated isomer with a trace of an isomer with an antennal PC/Fuc-modified LacdiNAc (see annotation in ***Supplementary Figure S1***). The *m/z* 1605 glycan is difucosylated $Man_6GlcNAc_2$; the spectrum below *m/z* 1500 (not shown) is dominated by a difucosylated *m/z* 1484 structure. Other data show the m/z 1484 and 1852 glycans in this fraction rapidly lose one hexose with jack bean α-mannosidase, indicative of a free α1,3-mannose and an antenna on the α1,6-arm.

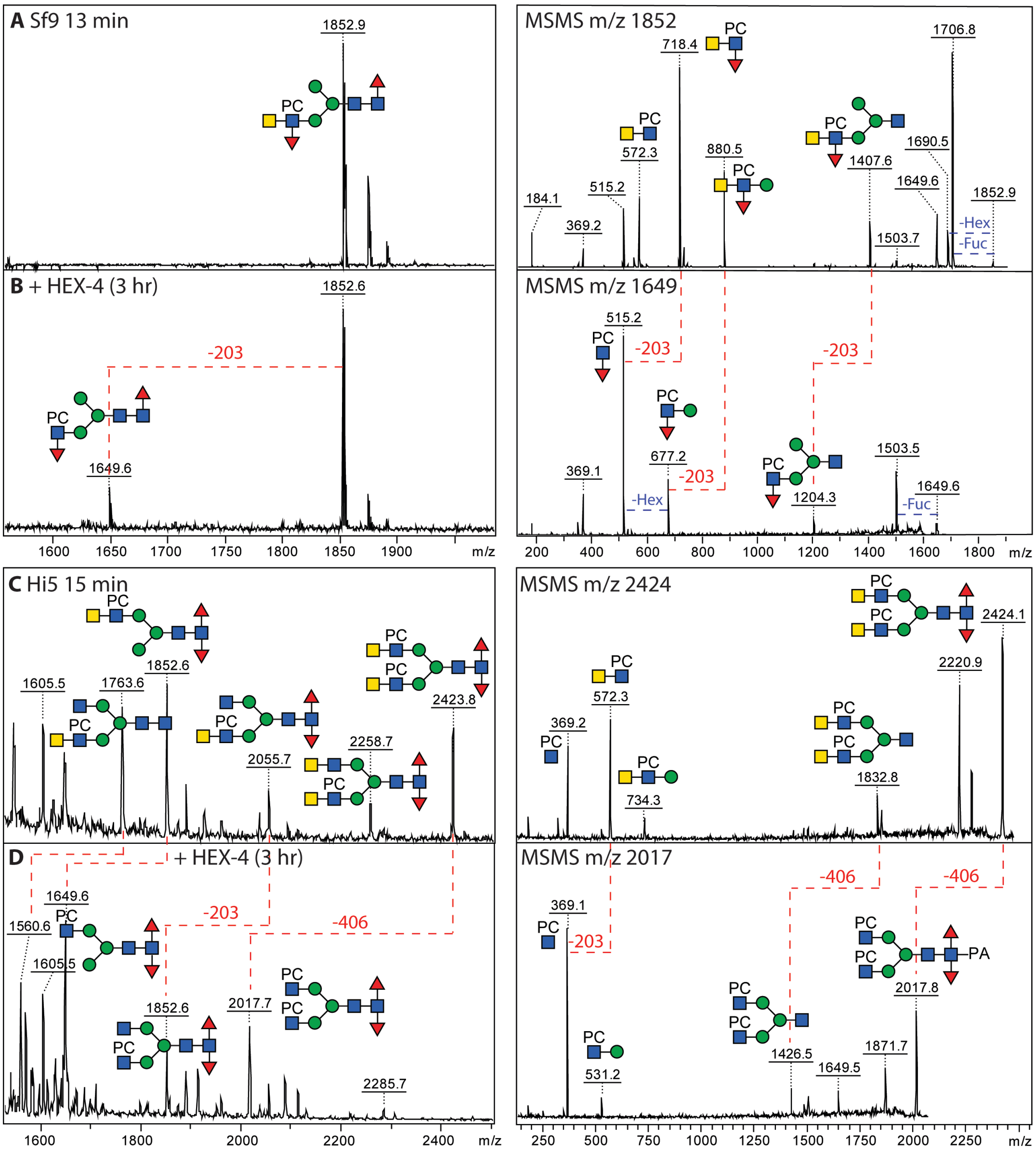

Supplementary Figure S3: Further characterisation of sulphated N-glycans from High Five cells. The anionic fractions eluting at 10 and 19 minutes on RP-amide (corresponding to 6 and 9.5 g.u.; see ***Supplementary Figure 1***) were subject to MALDI-TOF MS in negative modes with or without treatments with jack bean β-hexosaminidase, jack bean α-mannosidase or bovine α-fucosidase (A-F). Note that the α1,3-mannose is especially sensitive to the mannosidase and the unsulphated core α1,6-fucose, but not α1,3-fucose, is sensitive to the fucosidase. MS/MS spectra are shown for two *m/z* 1562 glycans and digestion products (G-K) as well as one *m/z* 1213 glycan (L). Three of the glycans in the 10 minute fraction are mannosidase sensitive (B); the combined fucosidase/mannosidase/hexosaminidase digest resulted in a single product for the *m/z* 1359, 1562 and 1765 glycans (C), indicative that the α1,6-mannose was sulphated rather than the core α1,6-fucose. The late-eluting *m/z* 1562 glycan was also fucosidase sensitive (E), but resistant to mannosidase (F), indicative that the α1,3-mannose was sulphated; in contrast, the *m/z* 1213 isomer was fucosidase insensitive, lost up to two mannose residues upon mannosidase treatment and possessed an intense *m/z* 225 fragment ion, indicative that the core α1,6-fucose was sulphated (L). The different positions for sulphation and resistance to hydrofluoric acid treatment have been shown for other insect glycans (Kurz et al., 2015; Cabrera et al., 2016; Stanton, et al., 2017; Hykollari, et al., 2019).

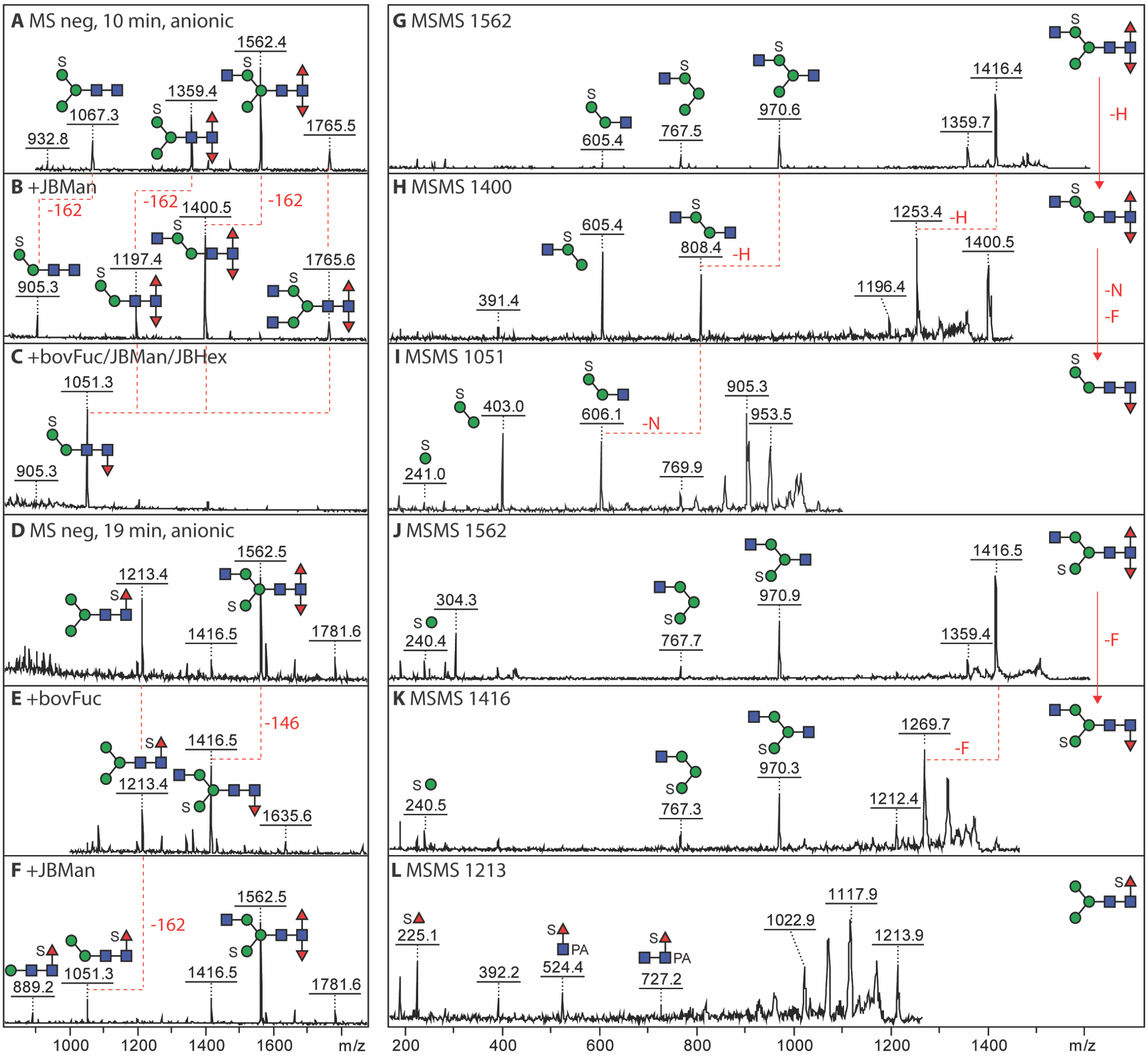

Supplementary Figure S4: Further characterisation of glucuronylated N-glycans from High Five cells. *(A-F)* The anionic fraction eluting at 12 minutes on RP-amide (see ***Supplementary Figure S1***) was subject to MALDI-TOF MS in positive (A-D) and negative (E-H) modes with or without treatment with jack bean β-hexosaminidase, human β-glucuronidase or hydrofluoric acid. Positive mode MS/MS spectra are shown for the *m/z* 2393 and 2597 glycans and their digestion products (I-P). While the *m/z* 2393 glycan is susceptible to both glycosidases, the *m/z* 2597 glycan (as well as that of *m/z* 2965) is resistant to β-glucuronidase and is the major remaining signal in negative mode within the shown mass range (F) indicating that the negative mode signal for these glycans is due to the glucuronic acid residue; considering the *m/z* 1113 fragment (M-O), it is proposed that this glycan carries a terminal HexNAc substitution of the glucuronic acid residue, akin to a motif previously found on insect N- and O-glycans (Gaunitz et al., 2013; Hykollari, et al., 2019; Kurz et al., 2015). The results of hydrofluoric acid treatment verify the presence of phosphorylcholine and a core α1,3-fucose residue (losses of 165 and 146 Da shown in green). After the removal of PC, B-fragments at *m/z* 542 and 745 are observed (L and P), corresponding to the major losses from the parent ions; subsequently the glucuronylated glycans can be digested down to *m/z* 1395 and 1541 with a deep-sea sediment metagenome-derived endo-β-*N*-acetylgalactosaminidase (NgaDssm; Sumida et al., 2024). For reasons of space, not all isomers (e.g., the *m/z* 2762 and its digestion products) are shown in all panels.

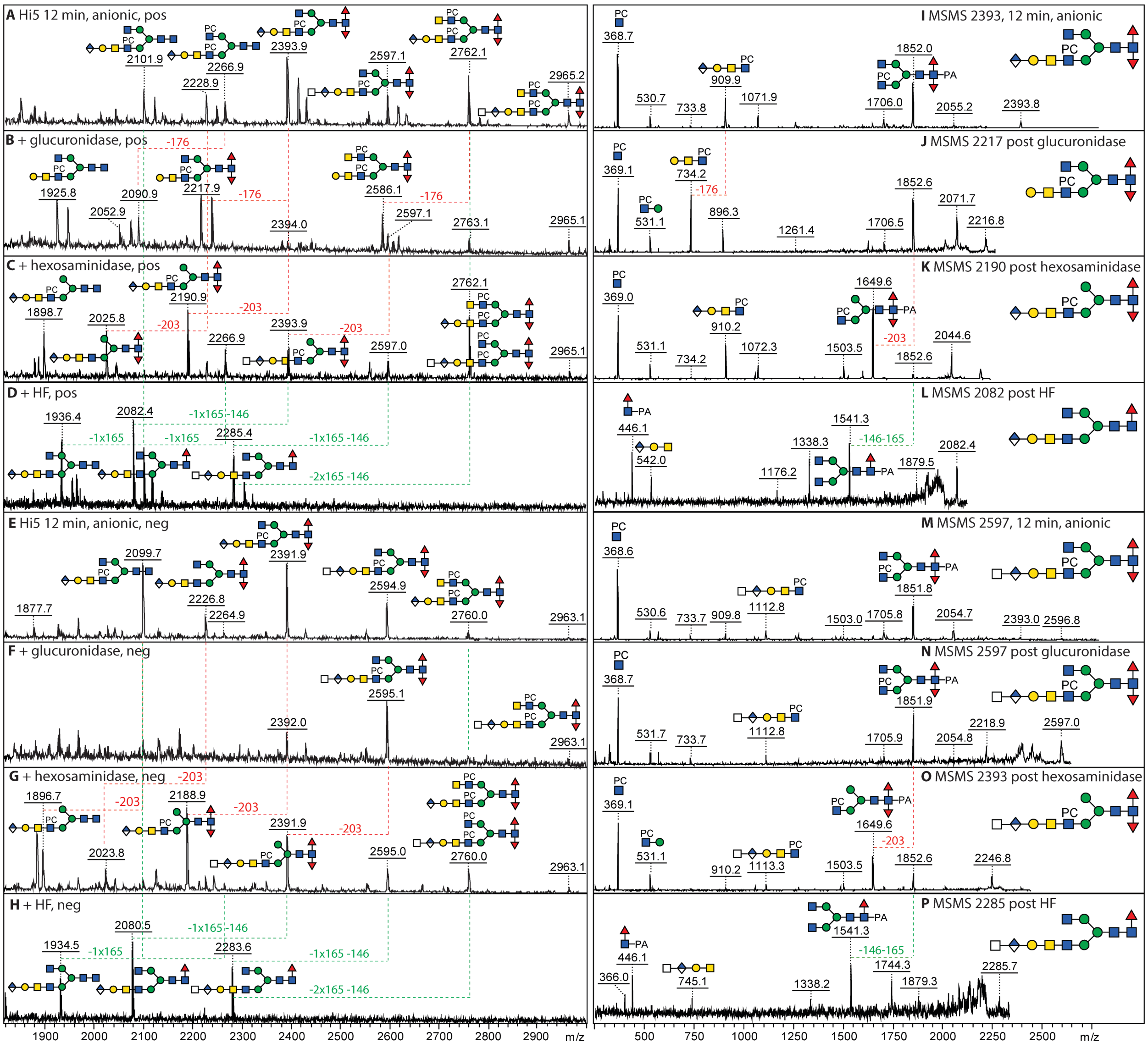

Supplementary Figure S5: MALDI-TOF MS analysis of unusual anionic N-glycans from Sf9 cells. The anionic subpool of N-glycans from Sf9 cells was subject to HPLC using an Ascentis RP-amide column. After screening by positive and negative ion mode MALDI-TOF MS, two selected fractions were subject to hydrofluoric acid treatment. The putative $Hex_3HexNAc_4Fuc_2HexA_1Pnt_{0-1}PC$ glycans were detected in both positive and negative modes (A, C, E and G); hydrofluoric acid treatment resulted in serial losses of phosphorylcholine and $Fuc_1HexA_1Pnt_{0-1}$ (B and F), whereby the intermediate products lacking one PC residue are well observed in negative mode (D and H), while the final products as $[M+Na]^+$ were dominant in the positive mode (B and F). MS/MS of $[M+H]^+$ parent ions was performed in either negative (I) or positive modes (J, L and M) for N-glycans in three RP-amide fractions (see also ***Figure 1*** in the main text or the mzxml files available via Glycopost), whereby the loss of the $Fuc_1HexA_1Pnt_{0-1}$ side chains from the parent ions correlates with the major $HexNAc_2PC_1Fuc_1HexA_1Pnt_{0-1}$ B ions at *m/z* 894 and 1026. MS/MS of the final hydrofluoric acid products as $[M+H]^+$ (K and N) indicated the replacement of these B-ion fragments by ones at *m/z* 407. The inset in panel K is a positive-mode MS/MS spectrum of the *m/z* 1863 intermediate hydrolysis product (corresponding to the *m/z* 1861 glycan detected in negative mode); the low intensity *m/z* 729 fragment may correspond to $HexNAc_2Fuc_1HexA_1$. Considering the HEX-4 sensitivity of the Sf9 *m/z* 1852 glycan (***Supplementary Figure S2***), it is presumed that the novel modifications of Sf9 cells are based on LacdiNAc (GalNAcβ1,4GlcNAc) modified with 6-linked PC and 3-linked fucose, substituted by $GlcA_1Xyl_{0-1}$, on the subterminal GlcNAc residue. The antennal modifications are indicated on the lower (α1,3) mannose, in keeping with the elution time of the probable *m/z* 1852 biosynthetic intermediate (7 g.u. or 15 minutes) and the fragmentation pattern. While hexuronic acid results in an approximately 7 minute shift to earlier elution time, pentose retards the retention time by 3 minutes. While the definition of hexuronic acid and pentose as glucuronic acid and xylose is based on the monosaccharides known in insects, the linkages are unknown, but preliminary data on another lepidopteran indicate sensitivity of the $PC/Fuc_1HexA_1Pnt_1$ epitope to *Sulfolobus* α-xylosidase. In contrast, the glucuronic acid containing motif in High Five cells is resistant to hydrofluoric acid, but sensitive to human β-glucuronidase (***Supplementary Figure S4***).

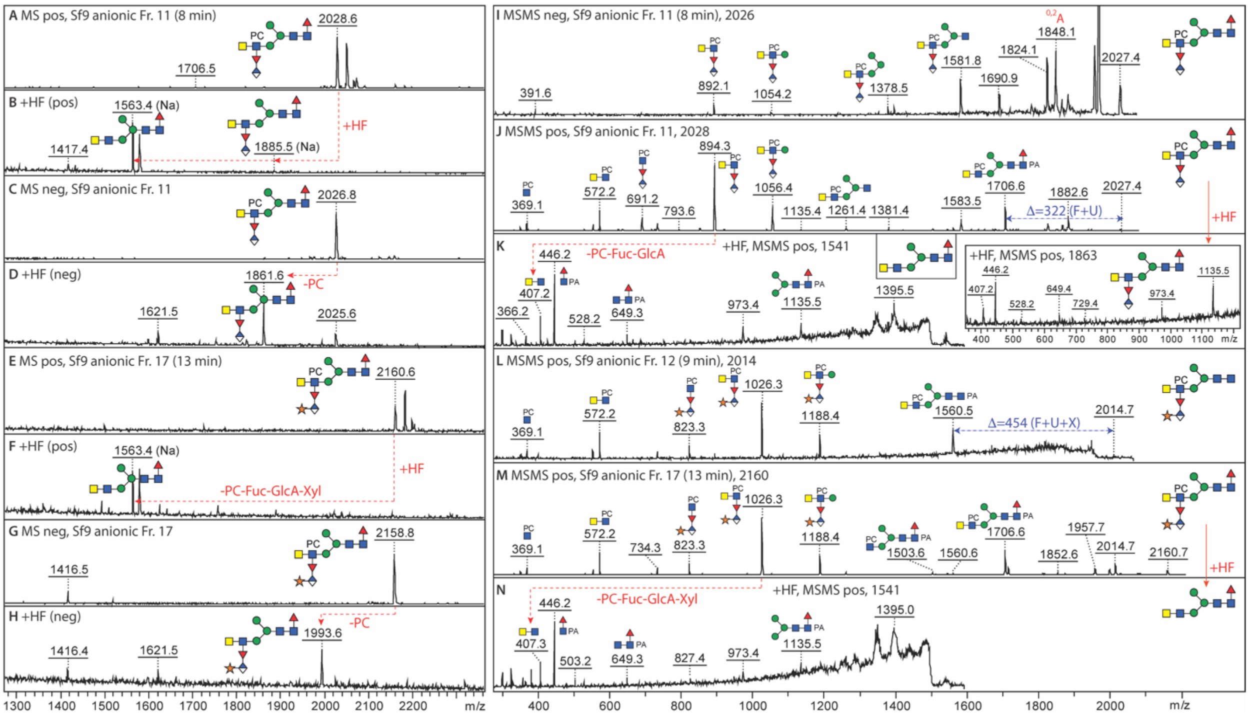

Supplementary Figure S6: Summary of modified LacdiNAc variations in High Five and Sf9 cells. Proposed structures of hybrid and complex N-glycans with LacdiNAc antennae - example structures with *m/z* values (for pyridylamino-labelled structures as $[M+H]^+$) and key Y ions are indicated (see red lines). The major *m/z* 1852 structure in *S. frugiperda* Sf9 cells has a PC/Fuc-modified LacdiNAc motif (also found in nematodes such as *Haemonchus contortus* and *Trichuris suis*), while the major form in *T. ni* High Five is core difucosylated. The further modifications diverge strongly as shown by MS/MS and different sensitivity to hydrofluoric acid (***Supplementary Figures S4 and S5***). In Sf9 cells the series of glycans is based on further modifications with a hexuronic acid and a pentose (presumed to be glucuronic acid and xylose) and would constitute a biosynthetic series; in HighFive cells, LacdiNAc is either modified with fucose and/or PC or with a glucuronidase-sensitive HexAHex motif (the same motif was previously defined by chemical/enzymatic digestion in *L. dispar* larvae). One glycan in HighFive cells (*m/z* 2908; ***Supplementary Figure S1***) was detected with one PC/Fuc-modified LacdiNAc on one antenna and one PC/HexAHex-modified LacdiNAc motif on the other; an as yet to be defined HexNAc can decorate the non-reducing terminal GlcA residues. The data suggest that fucosylation and glucuronylation/galactosylation are mutually exclusive modifications of LacdiNAc in *T. ni* cells - i.e., do not occur on the same antenna. Core difucosylation is dominant in High Five cells; additionally, sulphated glycans are found in the anionic pool. Sf9 cells only have trace amounts of core difucosylation with most processed structures possessing only core α1,6-fucosylation. The contrasting non-mammalian motifs with characteristic key Y-fragment ions should be considered when analysing glycoproteins produced in insect cell lines.

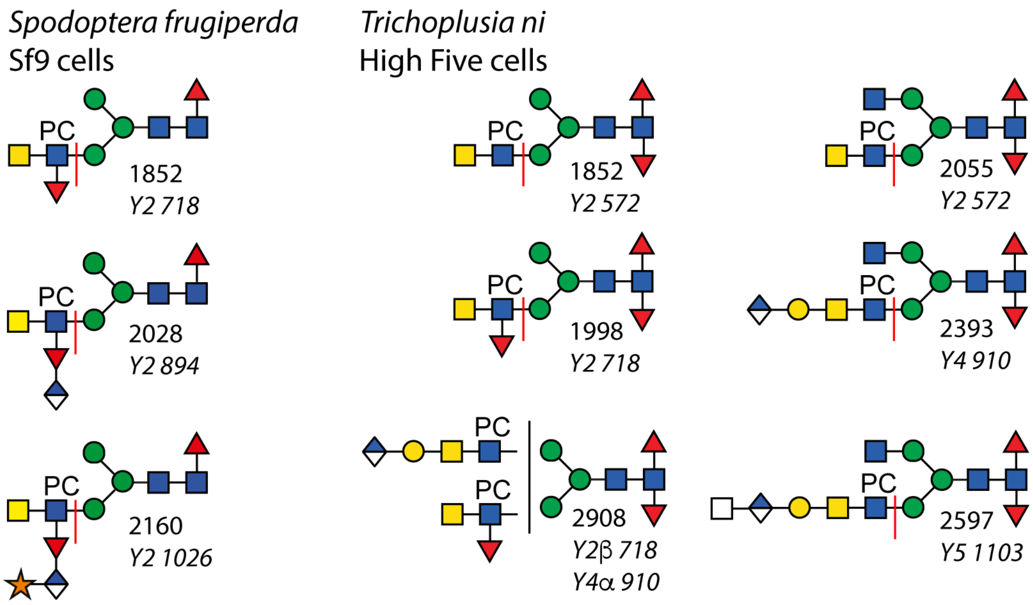

Supplemental Figure S7: Functional category enrichment analysis of insect cell glycoproteomes. Based on the LC-MS/MS glycoproteomics datasets, GO-term enrichment analysis (Fisher's Exact Test) was performed for glycoproteins bearing oligo-mannose- type N-glycans (inferred from glycan mass; compositions H5N2 to H9N2) of **(A)** Sf9 and **(B)** High Five cells. GO-term enrichment analysis of glycoproteins decorated with complex-type, i.e. not oligo-mannose-type) N-glycans of **(C)** Sf9 and **(D)** High Five cells. GO-term analysis was performed for the respective glycoprotein sets (i.e. test sets; blue bars) versus all protein sequences identified in this study as species specific reference sets (red bars) using Blast2Go (version 6.0.3 - build 202109151544; Götz et al, 2008).

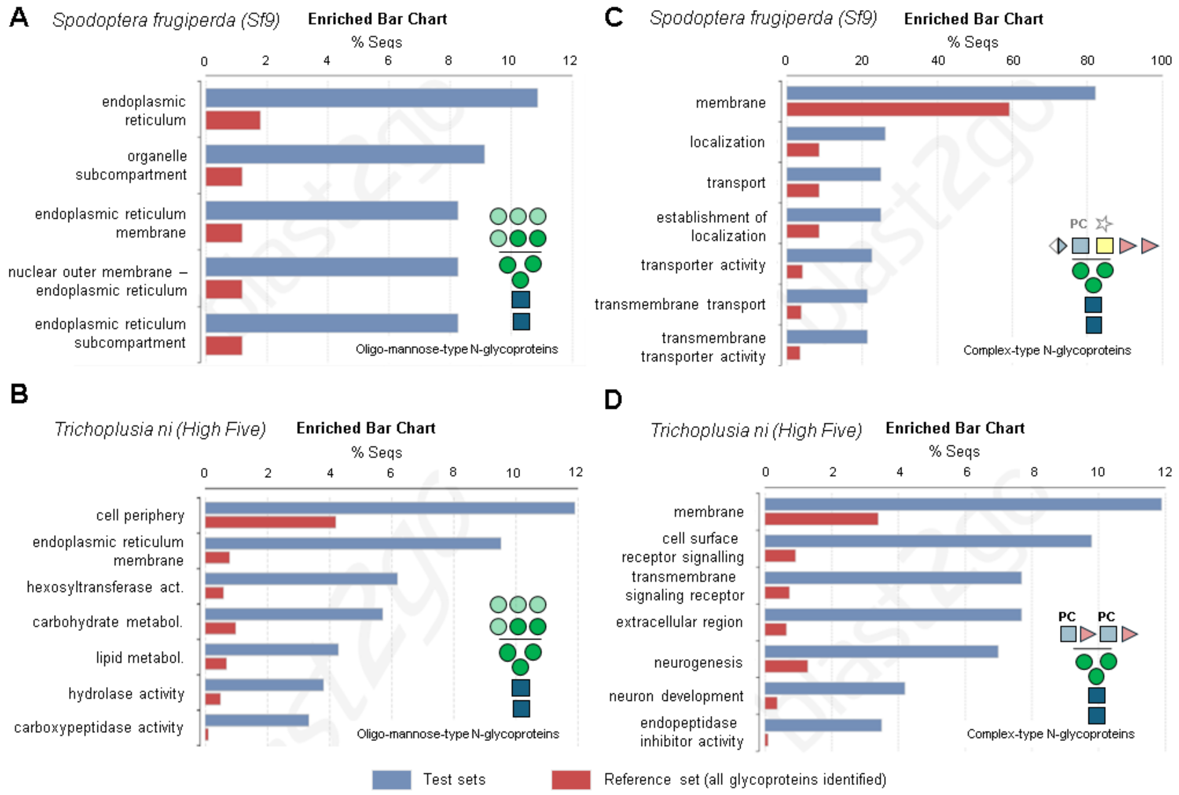

Supplementary Figure S8: MALDI-TOF MS of tryptic peptides and Western blotting of three commercial insect-derived recombinant influenza haemagglutinins. The A/California/04/2009, A/California/07/2009 and A/Victoria/361/2011 haemagglutinins were subject to SDS-PAGE and then to either tryptic peptide mapping or Western blotting. The near identity of the first two was verified by MALDI-TOF MS of the tryptic peptides, with the major difference being the occurrence of peptides of *m/z* 3804 or 3775 corresponding to amino acids 172-205 (difference of Thr or Ala). In the case of the A/Victoria/361/2011 haemagglutinin it was possible to assign two masses as glycopeptides on the basis of MS/MS: the peptides of *m/z* 2278 and 2481 are concluded to carry $Hex_3HexNAc_{2-3}Fuc_1$ N-glycans (see annotations in blue) on the peptide sequence NGTYDHDVYR (see annotations in red). MS signals found in all three samples are indicated with an asterisk; masses matching theoretical peptides for the corresponding Genbank protein sequence accessions are annotated with the corresponding amino acid numbers. Western blotting confirms the presence of phosphorylcholine epitopes on all three haemagglutinins as shown by binding to human C-reactive protein.

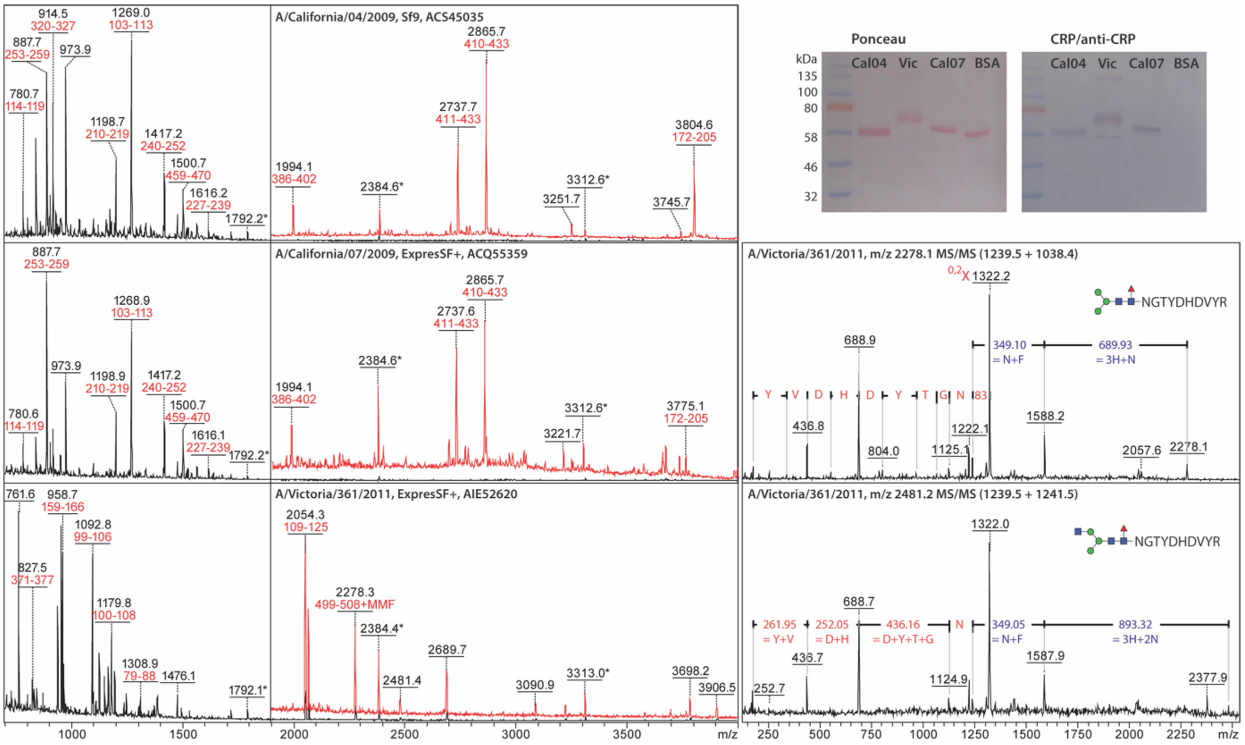

Supplementary Figure S9: MALDI-TOF MS of N-glycans of insect-derived recombinant influenza haemagglutinins. Mass spectra of the different HPLC fractions (see ***Figure 4*** of the main text); spectra are labelled as 'HA_sample_cell_RP-amide_retention-time', whereby Cal04_Tn, and Cal04_Sf correspond to A/California/04/2009, expressed either in a derivative of *T. ni* cells or in *S. frugiperda* Sf9 cells; Cal07_Ex and Vic361_Ex correspond to A/California/07/2009 and A/Victoria/361/2011 expressed in ExpresSF+ cells. For selected MS/MS, refer to ***Supplementary Figure S10.*** Western blotting of the two forms of A/California/04/2009 shows reactivity towards C-reactive protein and anti-HRP supporting the presence of antennal phosphorylcholine and core α1,3-fucose epitopes on a subset of N-glycans; the binding to C-reactive protein was abolished if the pentraxin was pre-incubated with EDTA (not shown). The higher number of α1,3-fucosylated glycans on *T. ni* cell-derived hemagglutinin as compared to the more obvious occurrence of phosphorylcholine on Sf9-derived samples correlate well with the Western blot data. Difucosylated and PC-modified glycans are highlighted with light pink or yellow boxes.

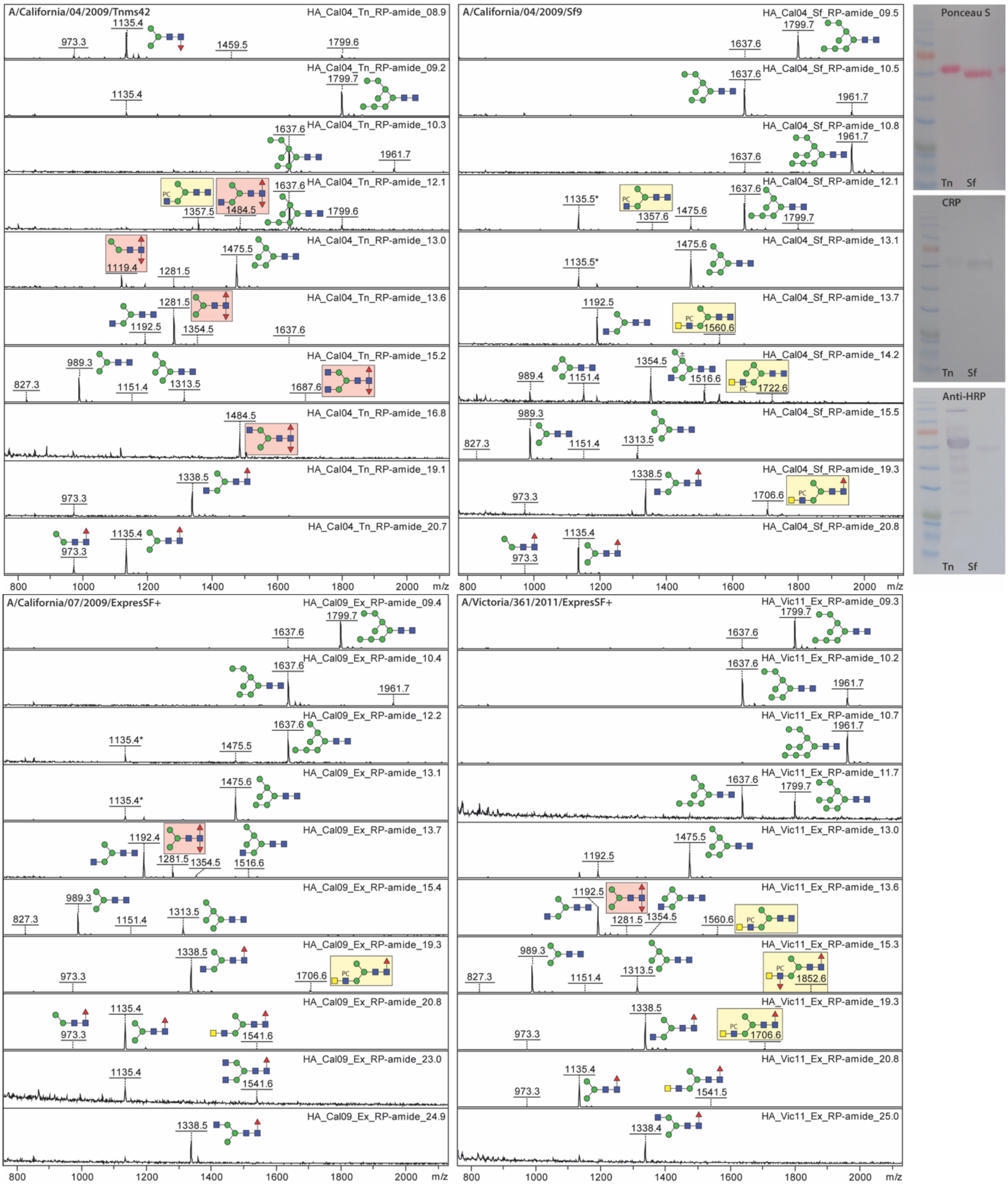

Supplementary Figure S10: MALDI-TOF MS/MS of N-glycans of insect-derived recombinant influenza haemagglutinins. The RP-amide HPLC chromatograms (upper panels) highlight the core fucosylated, LacdiNAc and PC-modified N-glycans; for the full annotations, refer to ***Figure 4*** of the main text. *(A-T)* Selected MS/MS spectra of structures present in the different HPLC fractions; glycans are labelled in the form 'HA_sample_cell_RP-amide_retention-time MSMS m/z', whereby Cal04_Tn, Cal04_Sf, Cal07_Ex and Vic361_Ex correspond to A/California/04/2009, expressed either in a derivative of *T. ni* cells or in *S. frugiperda* Sf9 cells and either A/California/07/2009 or A/Victoria/361/2011 expressed in ExpresSF+ cells. Key B and Y fragments are annotated, while overall structures are shown on the right.

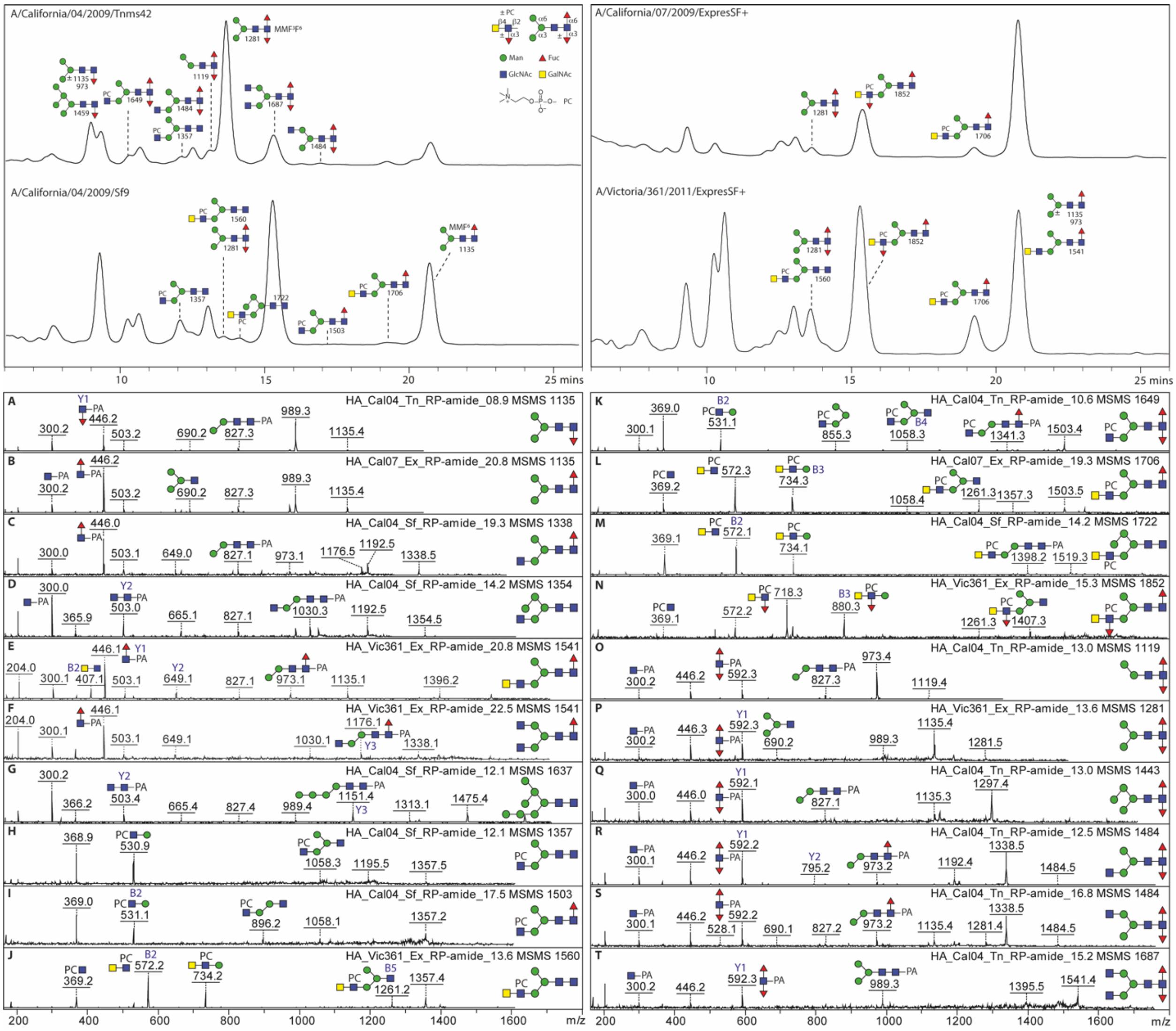

Supplementary Figure S11: LC-MS analysis of an Sf9-expressed haemagglutinin. Total ion chromatogram of the tryptic digest of A/California/04/2009 haemagglutinin (Genscript) from 15 to 37 min (A). Base peak extractions of oxonium ions at *m/z* 184.1 (B; PC_1), 351.1 (C; $HexNAc_1PC_1$-H_2O) and 369.1 (D; $HexNAc_1PC_1$), m/z 204.1 (E; $HexNAc_1$) and 366.2 (F; $HexNAc_1Hex_1$) highlighting the abundant presence of neutral and zwitterionic glycopeptides.

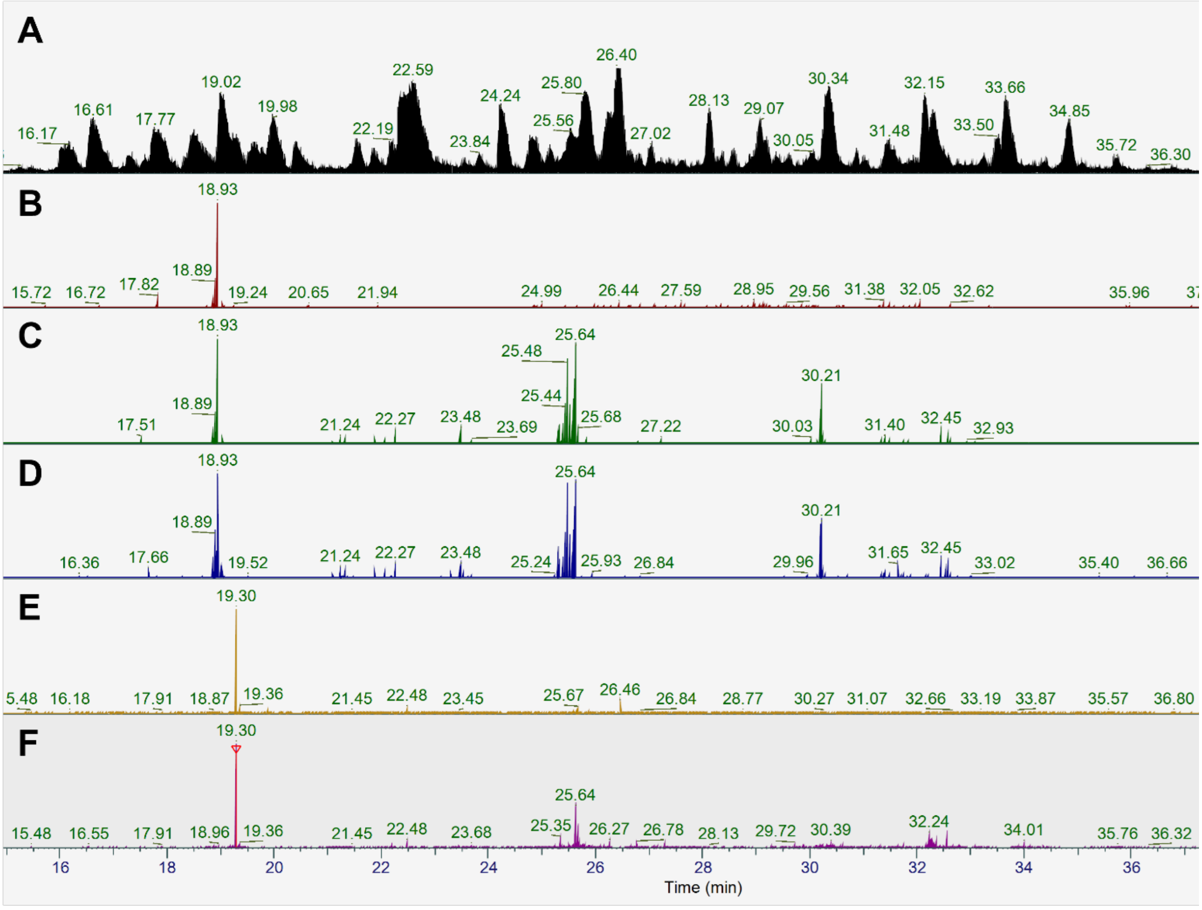

Supplementary Figure S12: Glycoproteomic analysis of Sf9-expressed A/California/04/2009 haemagglutinin N-glycopeptides carrying neutral glycans. A-D, MS/MS of haemagglutinin glycopeptide precursor ions at m/z 821.1 (3+ / A), 1304.7 (2+ / B), 1376.9 (3+ / C) and 1242.2 (3+ / D) corresponding to: NVTVTHSVLLEDK with $Hex_3HexNAc_2$ (A) or $Hex_3HexNAc_2Fuc_1$ (B) or NAGSGIIISDTPVHDCNTTCQTPK with $Hex_7HexNAc_2$ (C) or GAINTSLPFQNIHPITIGK with $Hex_8HexNAc_2$. Those glycopeptides (A-D) exhibited strong oxonium ions at *m/z* 204.1 (HexNAc), 366.1 ($HexNAc_1Hex_1$) and series of Y-peptide ions. For examples of phosphorylcholine-modified N-glycopeptides refer to Figure 5 in the main text.

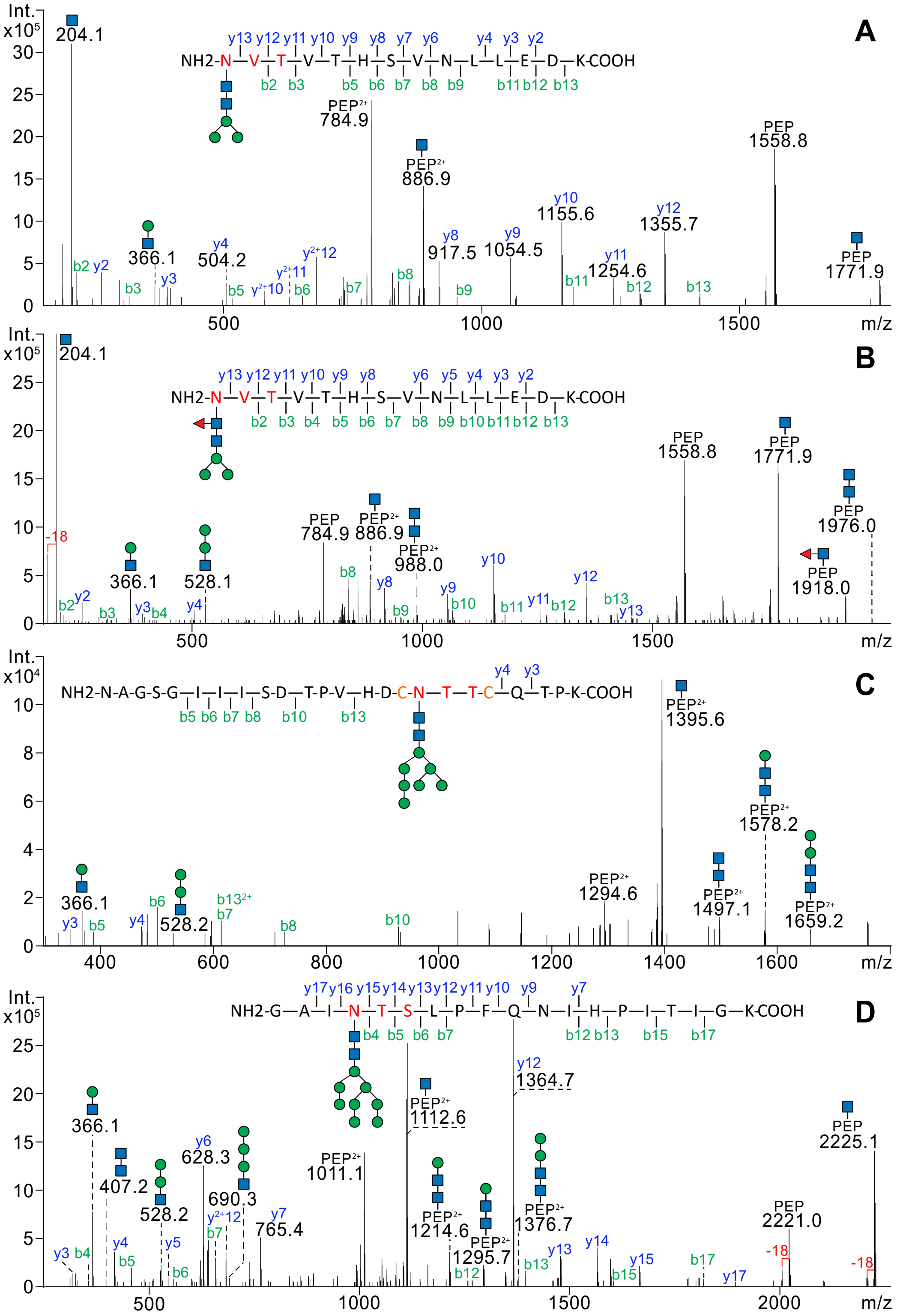

Supplementary Figure S13: Glycoproteomic analysis of Sf9-expressed A/California/04/2009 haemagglutinin N-glycopeptides. The sequence of the recombinant C-terminally His-tagged form of A/California/04/2009 haemagglutinin (see Uniprot accession) C3W5S1 with the signal sequence in italics, tryptic sites underlined and N-glycosylation sites in bold (those in red are proven by the current data). The extracted site-specific N-glycosylation is shown in terms of composition, additional mass and glycan type. Overall tryptic peptide coverage is estimated as 74.4% with two large peptides, including one predicted glycopeptide, not detected. Refer to the Supplementary Data File and files uploaded to the PRIDE database.

1 *MKAILVVLLY TFATANA*DTL CIGYHA**NNST** DTVDTVLEK**N VT**VTHSVNLL EDKHNGKLCK LRGVAPLHLG KCNIAGWILG
81 NPECESLSTA SSWSYIVETP SSD**N**GTCYPG DFIDYEELRE QLSSVSSFER FEIFPKTSSW PNHDSNKGVT AACPHAGAKS
161 FYKNLIWLVK KGNSYPKLSK SYINDKGKEV LVLWGIHHPS TSADQQSLYQ NADTYVFVGS SRYSKKFKPE IAIRPKVRDQ
241 EGRMNYYWTL VEPGDKITFE ATGNLVVPRY AFAMERNAGS GIIISDTPVH DC**NTT**CQTPK GAI**NTS**LPFQ NIHPITIGKC
321 PKYVKSTKLR LATGLRNIPS IQSRGLFGAI AGFIEGGWTG MVDGWYGYHH QNEQGSGYAA DLKSTQNAID EITNKVNSVI
401 EKMNTQFTAV GKEFNHLEKR IENLNKKVDD GFLDIWTYNA ELLVLLENER TLDYHDSNVK NLYEKVRSQL KNNAKEIGNG
481 CFEFYHKCDN TCMESVK**NGT** YDYPKYSEEA KLNREEIDGV KLESTRIYQH HHHHHH

N-Sites	N	H	Fuc	PC	GlcA	Mass	Type
NVT(40)	2	1				568.2116	Man-Based
	2	2				730.2644	
	2	3				892.3172	
	2	4				1054.3700	
	2	5				1216.4228	
	2	6				1378.4757	
	2	7				1540.5285	
	2	8				1702.5813	
	2	9				1864.6341	
	3	3				1095.3966	Complex
	2	1	1			714.2695	Fuc-based
	2	2	1			876.3223	
	2	3	1			1038.3751	
	3	3	1			1241.4545	
	3	3		1		1260.4521	PC-based
	4	3		1		1463.5314	
	4	3	1	1	1	1785.6214	
NTT(293)	2	3				892.3172	Man-Based
	2	4				1054.3700	
	2	5				1216.4228	
	2	6				1378.4757	
	2	7				1540.5285	
	2	8				1702.5813	
	2	9				1864.6341	
	3	3				1095.3966	Complex
	3	4				1257.4494	
	2	3	1			1038.3751	Fuc-based
	3	4	1			1403.5073	
	4	3		1		1463.5314	PC-based
	3	3	2	1		1552.5679	
	4	3	1	1		1609.5893	
	4	4		1		1625.5843	

N-Sites	N	H	Fuc	PC	GlcA	Mass	Type
NTS(304)	2	3				892.3172	Man-Based
	2	4				1054.3700	
	2	5				1216.4228	
	2	6				1378.4757	
	2	7				1540.5285	
	2	8				1702.5813	
	2	9				1864.6341	
	3	3				1095.3966	Complex
	3	4				1257.4494	
	3	5				1419.5022	
	2	3	1			1038.3751	Fuc-based
	3	4	1			1403.5073	
	4	3		1		1463.5314	PC-based
	4	4		1		1625.5843	
NGT(498)	2	1				568.2116	Man-Based
	2	2				730.2644	
	2	3				892.3172	
	2	4				1054.3700	
	2	5				1216.4228	
	2	6				1378.4757	
	2	7				1540.5285	
	2	8				1702.5813	
	2	1	1			714.2695	Fuc-based
	2	2	1			876.3223	
	2	3	1			1038.3751	
	2	4	1			1200.4279	
	3	3	1			1241.4545	
	4	3		1		1463.5314	PC-based
	4	3	2	1		1755.6473	

Supplementary Figure S14: MALDI-TOF MS of N-glycans of insect-derived recombinant SARS-CoV-2 Spike protein (Novavax). The RP-amide HPLC chromatogram highlights the core fucosylated, LacdiNAc and PC-modified N-glycans (refer to ***Figure 6*** of the main text for the full annotation and selected MS/MS). The mass spectra of the different HPLC fractions are labelled as 'Nov_RP-amide-retention-time'. The annotated *m/z* values are for $[M+H]^+$ in the positive ion mode (black) or $[M-H]^-$ in the negative ion mode (red); *m/z* values in italics are adducts or contaminants. The anti-horseradish peroxidase (anti-HRP), *Aleuria aurantia* lectin (AAL) and C-reactive protein (CRP) blots support the presence of core α1,3-fucose, core α1,6-fucose and phosphorylcholine epitopes on a subset of N-glycans on the recombinant Spike protein. Difucosylated and PC-modified glycans are highlighted with light pink or yellow boxes.

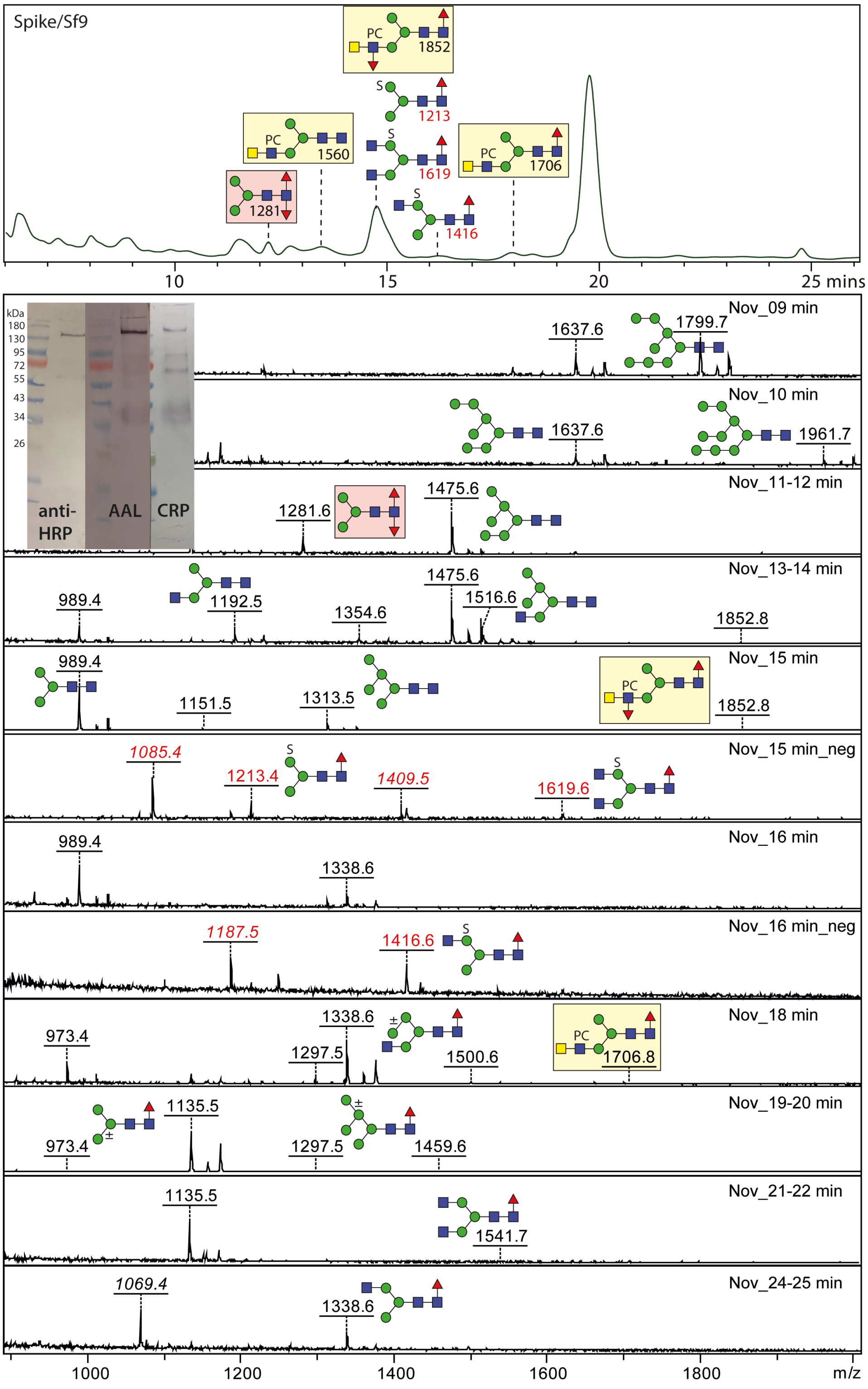

Supplementary Table 1. Compositions and theoretical *m/z* values for pyridylaminated lepidopteran N-glycans. Compositions are written as $H_xN_yF_{1\text{-}3}PC_{0\text{-}2}U_{0\text{-}1}X_{0\text{-}1}S_{0\text{-}1}$ (i.e., $Hex_xHexNAc_yFuc_{1\text{-}3}PC_{0\text{-}2}HexA_{0\text{-}1}Pnt_{0\text{-}1}S_{0\text{-}1}$, shown as, e.g., H3N6F2PC, whereby PC and S are phosphorylcholine and sulphate). Theoretical monoisotopic *m/z* were calculated by Glycoworkbench as $[M+H]^+$, except for sulphated glycans (in red) as $[M-H]^-$. For structures, also for glycans only in High Five cells > 2200 Da, refer to Supplementary Table 2. A tick indicates that the glycans were observed in the relevant glycoprotein or cellular sample by MALDI-TOF MS (see Figures 1, 4 and 6 or Supplementary Figures S1-S5, S9-S10 and S14).

Composition	*m/z*	High Five cells	Sf9 cells	Cal04 Tnms42	Cal04 Sf9	Cal07 ExpresSF	Vic361 ExpresSF	Spike Sf9
H1N2F	811.34							✓
H2N2	827.34	✓	✓					✓
H2N2F	973.39	✓	✓	✓	✓	✓	✓	✓
H3N2	989.39	✓	✓	✓	✓	✓	✓	✓
H3N2S	*1067.34*	✓						
H2N2F2	1119.46	✓		✓				
H3N2F	1135.45	✓	✓	✓	✓	✓	✓	✓
H4N2	1151.45	✓		✓	✓	✓	✓	✓
H3N3	1192.47	✓		✓	✓	✓	✓	✓
H3N2FS	*1213.39*	✓						✓
H3N3S	*1270.42*	✓						
H3N2F2	1281.51	✓	✓	✓	✓	✓	✓	✓
H4N2F	1297.50							✓
H5N2	1313.50	✓	✓	✓	✓	✓	✓	✓
H3N3F	1338.53	✓	✓	✓	✓	✓	✓	✓
H4N3	1354.52				✓		✓	✓
H3N3PC	1357.53	✓		✓	✓			
H3N2F2S	*1359.44*	✓						
H3N3FS	*1416.47*	✓						✓
H4N2F2	1443.56	✓						
H5N2F	1459.56		✓	✓				
H6N2	1475.55	✓	✓	✓	✓	✓	✓	✓
H3N3F2	1484.59	✓		✓				
H4N3F	1500.58							✓
H3N3FPC	1503.59		✓		✓			
H5N3	1516.58				✓		✓	
H3N4F	1541.61	✓	✓					
H3N4PC	1560.61	✓	✓					
H3N3F2S	*1562.52*	✓						
H5N2F2	1605.62	✓						
H3N4FS	*1619.55*	✓						✓
H7N2	1637.60	✓	✓	✓	✓	✓	✓	✓
H3N3F2PC	1649.64	✓		✓				
H3N4F2	1687.67	✓		✓				
H3N4FPC	1706.66	✓	✓		✓	✓	✓	✓
H4N4PC	1722.66				✓			
H3N5F	1744.64	✓						
H3N5PC	1763.66	✓						
H3N4F2S	*1765.61*	✓						
H8N2	1799.66	✓	✓	✓	✓	✓	✓	✓
H3N4F2PC	1852.72	✓	✓			✓	✓	✓
H3N4FPCU	1882.72		✓					
H4N4PCU	1898.69	✓						

H3N5FPC	1909.72	√						
H9N2	1961.71	√	√	√	√	√	√	√
H3N4F3PC	1998.77	√						
H3N4F1PCUX	2014.77		√					
H3N4F2PC2	2017.77	√						
H3N4F2PCU	2028.77		√					
H3N5F2PC	2055.80	√						
H4N5PCU	2101.77	√						
H10N2	2123.76	√	√					
H3N6PC2	2131.82	√						
H3N4F2PCUX	2160.81		√					
H4N4F2PCU	2190.83	√						

Supplementary Table 2. Compositions and theoretical *m/z* values for pyridylaminated and glycopeptide-linked lepidopteran N-glycans. Compositions are written as $H_xN_yF_{0\text{-}3}PC_{0\text{-}2}U_{0\text{-}1}X_{0\text{-}1}S_{0\text{-}1}$ (i.e., $Hex_xHexNAc_yFuc_{0\text{-}3}PC_{0\text{-}2}HexA_{0\text{-}1}Pnt_{0\text{-}1}S_{0\text{-}1}$, shown as, e.g., H3N6F2PC, whereby PC and S are phosphorylcholine and sulphate). Presence of a particular glycan mass in a sample is indicated by a tick (see Figures 1 and 2, Supplementary Figures S1-S6 and the text regarding explanations for the structural annotations). Theoretical *m/z* were calculated by Glycoworkbench as $[M+H]^+$, except for sulphated glycans as $[M-H]^-$ shown in red. For all compositions the mass difference for the corresponding peptide-bound glycan (see ***Figure 2*** in main text) is shown as well as relevant isomer-specific Glytoucan accessions. The sulphated and the largest glucuronylated N-glycans were not detected in the glycoproteomic analysis.

Composition	*m/z* *PA-glycan*	*Δm/z* *glycan-H₂O*	Glytoucan accession	High Five glycans	High Five glycopep	Sf9 cells	Sf9 glycopep
H2N2 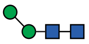	827.34	730.27	G22573RC	✓	✓	✓	✓
H2N2F 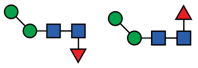	973.39	876.32	G83878UZ G00395TQ	✓	✓	✓	✓
H3N2 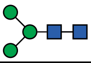	989.39	892.32	G22768VO	✓	✓	✓	✓
H3N2S 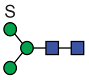	*1067.34*	972.27	G04724RA	✓			
H2N2F2 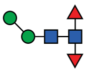	1119.46	1022.39	G05890XZ	✓	✓		
H3N2F 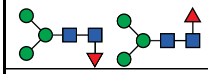	1135.45	1038.38	G92612ZM G45995IV	✓	✓	✓	✓
H4N2 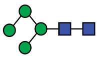	1151.45	1054.37	G09724ZC	✓	✓		✓
H3N3 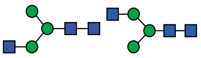	1192.47	1095.40	G70073SG G06920GM	✓	✓	✓	✓
H3N2FS 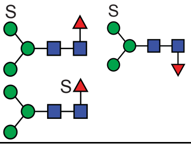	*1213.39*	1118.32	G51851AH G50009AO G34964EN	✓			
H3N3S 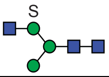	*1270.42*	1175.35	G62101RX	✓			
H3N2F2 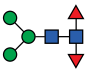	1281.51	1184.44	G77479VH	✓	✓	✓	
H4N2F 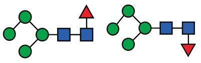	1297.50	1200.43	G48823SZ G76791LV	✓			
H5N2 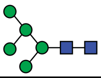	1313.50	1216.43	G55220VL	✓	✓	✓	✓
H3N3F 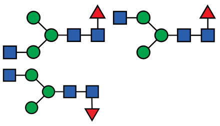	1338.53	1241.46	G14576KZ G69987TD G48288NV	✓	✓	✓	✓

H4N3<br>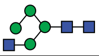	1354.45	1257.38	G53168IY			✓	
H3N3PC<br>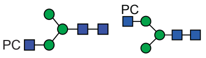	1357.53	1260.46	G80096FU<br>G88989KF	✓	✓		
H3N2F2S<br>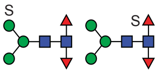	*1359.44*	1266.37	G05021DD<br>G48254JX	✓			
H3N3FS<br>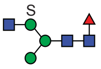	*1416.47*	1321.40	G10001NM	✓			
H4N2F2<br>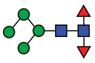	1443.56	1345.49	G26594WY	✓			
H5N2F<br>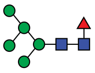	1459.56	1362.49	G99858XP			✓	
H6N2<br>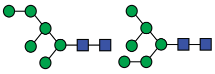	1475.55	1378.48	G61846BY<br>G80966KZ		✓	✓	✓
H3N3F2<br>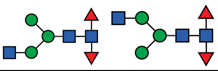	1484.59	1387.52	G41720HN<br>G10425SO	✓		✓	
H4N3F<br>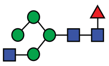	1500.58	1403.51	G58969RU			✓	
H3N3FPC<br>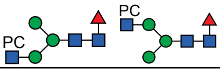	1503.59	1406.52	G71395YL<br>G73657JW	✓		✓	✓
H5N3<br>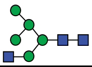	1516.57	1419.50	G08520NM			✓	
H3N4F<br>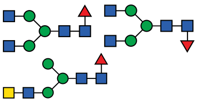	1541.61	1444.54	G80858MF<br>G20956ZK<br>G20956ZK	✓		✓	
H3N4PC<br>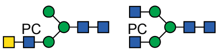	1560.61	1463.54	G16427JQ	✓		✓	✓
H3N3F2S<br>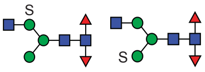	*1562.52*	1467.45	G44752TZ<br>G39252LC	✓			
H5N2F2<br>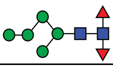	1605.62	1508.55	G17458HS	✓			
H3N4FS<br>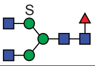	*1619.55*	1524.48	G50977NL	✓			
H7N2<br>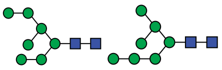	1637.60	1540.53	G83161QT<br>G68668TB	✓	✓	✓	✓
H3N3F2PC<br>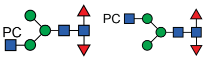	1649.64	1552.57	G34964XD<br>G13503UO	✓	✓		
H5N3F<br>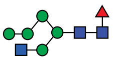	1662.63	1565.56	G82371GR	✓			

H3N4F2 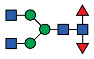	1687.67	1590.60	G69912LP	✓				
H3N4FPC 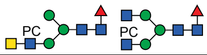	1706.66	1609.59	G16427JQ<br>G56477EX	✓	✓	✓	✓	
H4N4PC 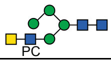	1722.66	1625.59	G74626KE			✓		
H3N5F 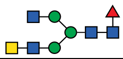	1744.67	1647.60	G88461FF	✓				
H3N5PC 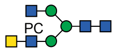	1763.66	1666.59	G83310WI	✓				
H3N4F2S 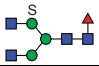	*1765.60*	1670.53	G15159WT	✓				
H8N2 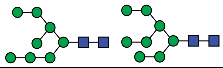	1799.66	1702.59	G40702W<br>G89864BN	✓	✓	✓	✓	
H3N4F2PC 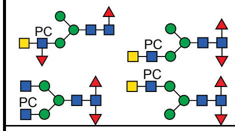	1852.72	1755.65	G13755PW<br>G15030NM<br>G04565CX<br>G48199XW	✓	✓	✓	✓	
H3N4FPCU 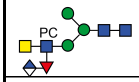	1882.72	1785.64	G46340KS			✓	✓	
H4N4PCU 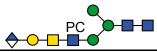	1898.69	1801.62	G17988GM	✓				
H3N5FPC 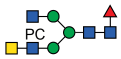	1909.72	1812.65	G82370HC	✓				
H9N2 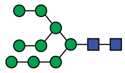	1961.71	1864.64	G60230HH	✓	✓	✓	✓	
H3N4F3PC 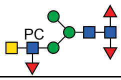	1998.77	1901.70	G88572EA	✓				
H3N4F1PCUX 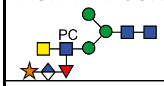	2014.77	1917.69	G23875AR			✓		
H3N4F2PC2 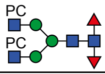	2017.77	1920.70	G87492HF	✓				
H3N4F2PCU 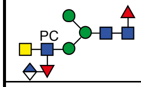	2028.77	1931.69	G74462VM			✓	✓	
H3N5F2PC 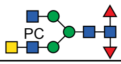	2055.80	1958.73	G29392CO	✓				
H4N5PCU 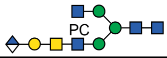	2101.77	2015.73	G83398PN	✓				
H10N2 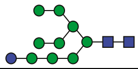	2123.76	2026.69	G19958IL	✓	✓	✓	✓	
H3N6PC2 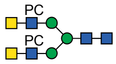	2131.82	2034.75	G17444QF	✓				

H3N4F2PCUX<br>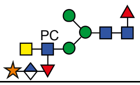	2160.81	2063.73	G94108VA			✓	✓
H4N4F2PCU<br>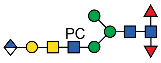	2190.83	2093.76	G17395MT	✓			
H3N5F3PC<br>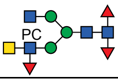	2201.85	2104.78	G14418ZB	✓			
H3N5F2PC2<br>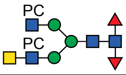	2220.85	2123.78	G07705RQ	✓			
H4N5F2U<br>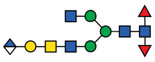	2228.86	2131.79	G02792GJ	✓			
H4N5FPCU<br>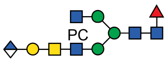	2247.82	2150.75	G72160RQ	✓			
H3N6F2PC<br>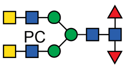	2258.88	2161.81	G70763UN	✓			
H4N5PC2U<br>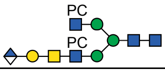	2266.83	2169.76	G76286CY	✓			
H3N6FPC2<br>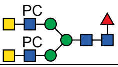	2277.87	2180.80	G80597BE	✓			
H11N2<br>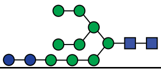	2285.81	2188.74	G18948TG	✓			
H3N5F3PC2<br>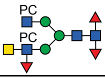	2366.91	2269.84	G14318VU	✓			
H4N5F2PCU<br>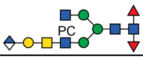	2393.92	2296.85	G57311KM	✓			
H3N6F2PC2<br>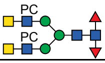	2423.93	2326.86	G72903DW	✓			
H4N6PC2U<br>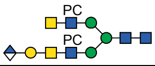	2469.88	2372.81	G95490ED	✓			
H4N5F2PC2U<br>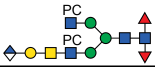	2558.97	2461.90	G24710QX	✓			
H4N6F2PCU<br>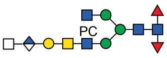	2597.01	2499.94	G64182PL	✓			
H4N6F2PC2U<br>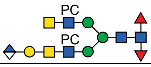	2762.01	2664.94	G53524GH	✓			
H4N6F3PC2U<br>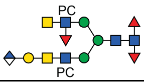	2908.06	2810.99	G27872BU	✓			
H4N7F2PC2U<br>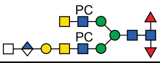	2965.09	2868.02	G86812FB	✓			

Supplementary Table 3. Summary of analytical methods and data for recombinant viral glycoproteins. Four recombinant influenza haemagglutinins and one recombinant SARS-CoV-2 Spike were analysed by a variety of LC, MS, MS/MS and Western blotting techiques: A/California/04/2009 (expressed in either *T. ni* Tnms42 cells or *S. frugiperda* Sf9 cells), A/California/07/2009 and A/Victoria/361/2011 (produced in *S. frugiperda* ExpresSF+ cells) and Nuvaxovid (expressed in Sf9 cells).

**Method**	**Cal04 Tnms42**	**Cal04 Sf9**	**Cal07 ExpresSF**	**Vic361 ExpresSF**	**Spike Sf9**
Tryptic digest		Suppl. Fig. 8	Suppl. Fig. 8	Suppl. Fig. 8	
LC-MALDI-MS (PA-glycans)	Figure 4 Suppl. Fig. 9	Figure 4 Suppl. Fig. 9	Figure 4 Suppl. Fig. 9	Figure 4 Suppl. Fig. 9	Figure 6
MALDI-MS/MS (PA-glycans)	Suppl. Fig. 10	Suppl. Fig. 10	Suppl. Fig. 10	Suppl. Fig. 10	Figure 6 Suppl. Fig. 14
LC-ESI-MS/MS (glycopeptides)		Figure 5 Suppl. Fig. 11-13			
CRP (Western blot)	Suppl. Fig. 9	Suppl. Fig. 8/9	Suppl. Fig. 8	Suppl. Fig. 8	Suppl. Fig. 14
Anti-HRP (Western blot)	Suppl. Fig. 9	Suppl. Fig. 9			Suppl. Fig. 14
AAL (Western blot)					Suppl. Fig. 14
